# Integrated spatial analysis and Gaussian dispersion modelling of airborne particulate matter from rice processing mill hubs in rural Bangladesh and associated public health risks

**DOI:** 10.1007/s11356-026-38036-9

**Published:** 2026-07-22

**Authors:** Rubaiatul Islam Zerin, Md. Kamrul Hossain, Humaira Rashid, Sababa Tasnim, Md. Julfikar Ali, Md. Mizanur Rahman, Mohd. Maniruzzaman, Rafiquel Islam

**Affiliations:** 1 Department of Environmental Science and Geography, Islamic University, Kushtia, 7003 Bangladesh; 2 Department of Environmental Science and Management, Independent University, Dhaka, 1245 Bangladesh; 3 Department of Information and Communication Technology, Islamic University, Kushtia, 7003 Bangladesh; 4 Department of Mathematics, Faculty of Science, Islamic University, Kushtia, 7003 Bangladesh; 5 Department of Applied Chemistry and Chemical Engineering, Islamic University, Kushtia, 7003 Bangladesh; 6 School of Environmental and Life Sciences, The University of Newcastle, Callaghan, NSW 2308 Australia; 7 Applied Research Center for Environment and Marine Studies, King Fahd University of Petroleum & Minerals, Dhahran 31261, Saudi Arabia

**Keywords:** Rice mills, Air particulate matter, Dispersion modeling, Human health risks, AirQ + tool

## Abstract

The rapid expansion of rice processing mills in agriculturally rich regions, such as Khajanagar, Kushtia, has resulted in elevated concentrations of particulate matter (PM), posing serious environmental and public health risks. The close proximity of rice mills to residential areas necessitates evaluating particulate matter dispersion to assess neighbourhood-level health risks. This study examines the spatial distribution, dispersion behaviour, and health impacts of PM₂.₅ and PM₁₀ across industrial and adjacent residential zones. A total of 69 sampling points were monitored using optical particle counters (OPCs) at 0–100 m (industrial) and 101–300 m (residential) distances, along with meteorological observations. PM₂.₅ concentrations in industrial areas exceeded all regulatory limits in Bangladesh, the WHO, and the U.S. EPA, while PM₁₀ exceeded WHO standards only within industrial zones; both fractions exceeded all benchmarks in residential areas. The Kruskal–Wallis test (*p* < 0.05) confirmed significant interzonal variation. Spatial mapping identified PM hotspots in the western and eastern industrial belts and the western residential cluster, providing empirical evidence of localized emission intensity and downwind accumulation driven by mill density and prevailing wind patterns. PM₂.₅ and PM₁₀ were highly correlated, though their relationships with humidity and temperature were weak. Gaussian plume modelling indicated that PM₁₀ dispersed and deposited over greater distances than PM₂.₅ due to its size-dependent transport under prevailing winds, increasing exposure in residential zones. AirQ⁺ health risk analysis revealed that PM₂.₅ exposure contributed to 39.78% of ALRI-related mortality in children under five, while in adults (≥ 30 years), it accounted for 48.01% of Chronic Obstructive Pulmonary Disease (COPD), 56.25% of Ischemic Heart Disease (IHD), and 65.05% of stroke deaths. PM₁₀ posed additional risks for IHD and lung cancer, with 34.16% of lung cancer mortality linked to PM₂.₅ exposure. By integrating spatial monitoring, dispersion modelling, and health risk assessment, this study identifies high-risk micro-environments and underscores the need for stricter emission control in rice-processing regions.

**Graphical abstract:**

**Figure:**
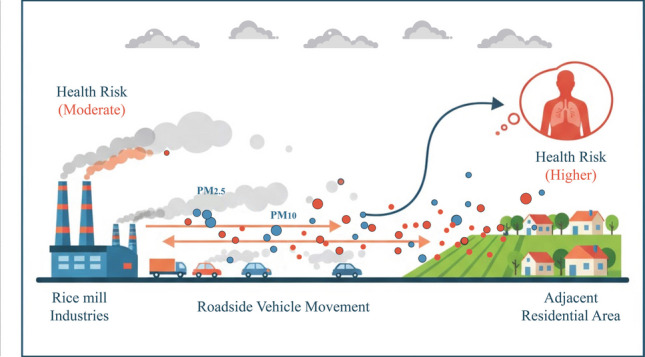

**Supplementary Information:**

The online version contains supplementary material available at 10.1007/s11356-026-38036-9.

## Introduction

Rice is one of the most important staple foods in the world, supporting numerous economies, while most of the world's rice is produced and consumed in developing nations. However, while paddy is harvested from the fields and processed into rice, this process contaminates the environment by producing air particulate matter (APM), as these rice processing technologies have not changed significantly to adopt an eco-friendly approach. In large-scale rice processing, husking is done in traditional rice mills in Bangladesh. Most rice mills lack remediation-based chimneys or other protective measures. Rice milling wastes are often burned, contributing to atmospheric carbon levels and impacting the ozone layer. Large quantities are dumped, creating disposal problems, releasing particulate matter and methane, and degrading the environment in rice-producing areas. In recent times, some districts, such as Nagaon, Chapa Nawabganj, Dinajpur, Kushtia, and Noapara of Jessore, have invested in establishing large automatic rice mills (Rahman et al. 2017). On an average, Bangladesh has about 15,500 husking mills, 650 semi-automatic mills, and 300 automatic rice mills (Bhuiyan et al. 2022).

Air pollution concentrations vary significantly by land use, with higher levels in transportation corridors, commercial and industrial zones, and lower levels in residential areas, indicating strong spatial heterogeneity driven by emission sources (Lee et al. 2025). Previous studies have consistently shown that particulate matter (PM) concentrations are generally higher in urban and industrial environments than in purely rural areas (Hsu et al. 2021). Studies in industrial areas of Nigeria report elevated levels of PM_2.5_ and PM_10_ linked to emissions from industrial stacks and processing activities. These concentrations often exceed WHO guideline values and are higher near emission sources, indicating strong spatial gradients. Such pollution has been associated with potential respiratory health risks for nearby populations (Ayua et al. 2020, 2023). However, recent evidence suggests that agricultural-related industrial activities, particularly rice milling and rice husk processing, can act as significant sources of fine particulate matter (PM₂.₅), generating localised pollution hotspots even outside major urban centres (Ansari et al. 2017). Recent studies demonstrate that particulate matter emissions from agro-industrial activities, particularly rice straw burning and rice husk combustion, are not confined to local scales but exhibit significant spatial dispersion influenced by meteorology and source intensity. Evidence from Vietnam and Thailand shows that rice-related biomass burning generates substantial PM_2.5_ and PM_10_ emissions that exceed WHO guidelines and affect surrounding rural and urban environments (Le et al. 2022; Suriyawong et al. 2023). Despite this growing recognition, the extent to which emissions from agricultural processing industries influence air quality in surrounding non-agricultural areas, such as nearby industrial zones and residential settlements, remains poorly understood. In particular, limited information is available on the spatial dispersion patterns and deposition behaviour of PM originating from mixed sources, including both agricultural and non-agricultural activities. To address this knowledge gap, this study explicitly investigates the impact of rice husk–based agricultural processing emissions on particulate matter distribution in adjacent non-agricultural areas. Gaussian Dispersion Modelling (GDM) is employed to simulate the transport, diffusion, and deposition of PM using site-specific meteorological and emission data. This approach enables the characterisation of pollutant dispersion beyond source locations and provides insight into the probability and spatial extent of PM exposure in surrounding industrial and residential environments (Alemayehu and Hackett 2015).

Rice milling also poses a significant occupational health threat. Labour-intensive activities, such as load carrying, boiler firing, rice drying, and machine operation, are usually conducted under unfavourable working conditions (Siddique 2007). The risks associated with exposure to fine particulate matter (PM_2.5_) are significant, as it penetrates deep into the lungs, increasing the risk of respiratory infections, asthma, chronic obstructive pulmonary disease (COPD), cardiovascular disease, and lung cancer (Cohen et al. 2017; Chanunya Srihawan 2019). The larger particles (PM_10_) can cause bronchitis, worsen asthma, and impair lung function, particularly in occupational settings with high dust levels (Liu et al. 2019). There has also been a record of prevalence of musculoskeletal disorders and respiratory dysfunctions, skin and eye irritation, and heat-related illnesses among the workers in rice mills (Siddique 2007; Eshwaramma et al. 2016). Such risks are exacerbated by poor working conditions, including the lack of protective gear, overwork, and poor health care.

Air pollution is a key global health issue. It caused about 6.67 million deaths in 2019, accounting for almost 12 percent of total mortality (Di et al. 2017). Exposure to particulate matter is closely linked to disease burden, and children aged under five are especially susceptible to Acute Lower Respiratory Infection (ALRI), and adults have a high mortality rate owing to stroke, ischemic heart disease (IHD), COPD, and lung cancer. A study conducted in large industrial cities of Bangladesh, such as Dhaka, Narayanganj, and Gazipur, indicates that PM_2.5_ and PM_10_ are frequently measured above WHO standards (Hossain et al. 2026). Wind direction and speed are strongly linked to pollutant dispersion, and Hazard Quotient studies indicate a high non-carcinogenic risk, particularly from exposure to PM_2.5_. The results emphasise that the lack of control over emissions, the need for tougher restrictions, and specific actions to reduce risks to the population's health in industrial areas are extremely important. The vital role of small-scale rice mills has also been suggested in recent studies as a contributor to local PM_2.5_ levels in semi-urban Bangladesh (Hasan et al. 2025). Despite extensive research on rice productivity and profitability in the country to meet the demand of the growing population, a significant knowledge gap persists concerning the negative impacts of particulate matter emitted from the rice processing industries, which has not been investigated yet, nor created health issues for the workers and the surrounding communities due to the dispersion of this particulate matter.

In particular, the current research focuses on Khajanagar, in Kushtia, one of Bangladesh’s major rice-processing and wholesale hubs. The area hosts approximately 450 rice husking and processing mills (Kudrote Khuda Sobuj 2023), which generate particulate matter, including PM_2.5_ and PM_10_, exposing workers and nearby residents to hazardous dust concentrations. Although previous studies have assessed health risks and the dispersion of airborne particulate matter (PM) in a fragmented manner, no research has specifically focused on identifying spatial hotspots of PM from rice processing industries in climate-driven, environmentally vulnerable countries like Bangladesh. Still, to date, no studies have focused on this territory, evaluating the contamination status by PMs and their model-based dispersion behaviour, and associated specific diseases like Acute Lower Respiratory Infections (ALRI) in children and chronic diseases like lung cancer, Chronic Obstructive Pulmonary Disease (COPD), and ischemic heart disease in adults, using the AirQ + tool developed by WHO. So, this study aims to: (1) Determine existing levels of PM_2.5_ and PM_10_ and their spatial distribution; (2) Examine the Gaussian Dispersion Modelling-based distributions of particulate matter from agro-industry and vehicular emission, and deposition status in the surrounding residential area; and (3) Determine possible health risks and disease-specific attributable cases of the long-term effects of exposure in both industrial and residential areas through the AirQ + tool. The results will provide valuable insights into the impact of pollution originating from rice mills and roadways on the surrounding ecosystem and human health. This research will enhance stakeholders' awareness of air pollution hazards from rice production and assist policymakers and environmental agencies in developing sustainable strategies for this type of industrial and agricultural planning. Finally, this research will promote ecologically sustainable practices and protect public health, aligning with Bangladesh's sustainable development objectives.

## Materials and methods

### Study area

**Fig. 1:**
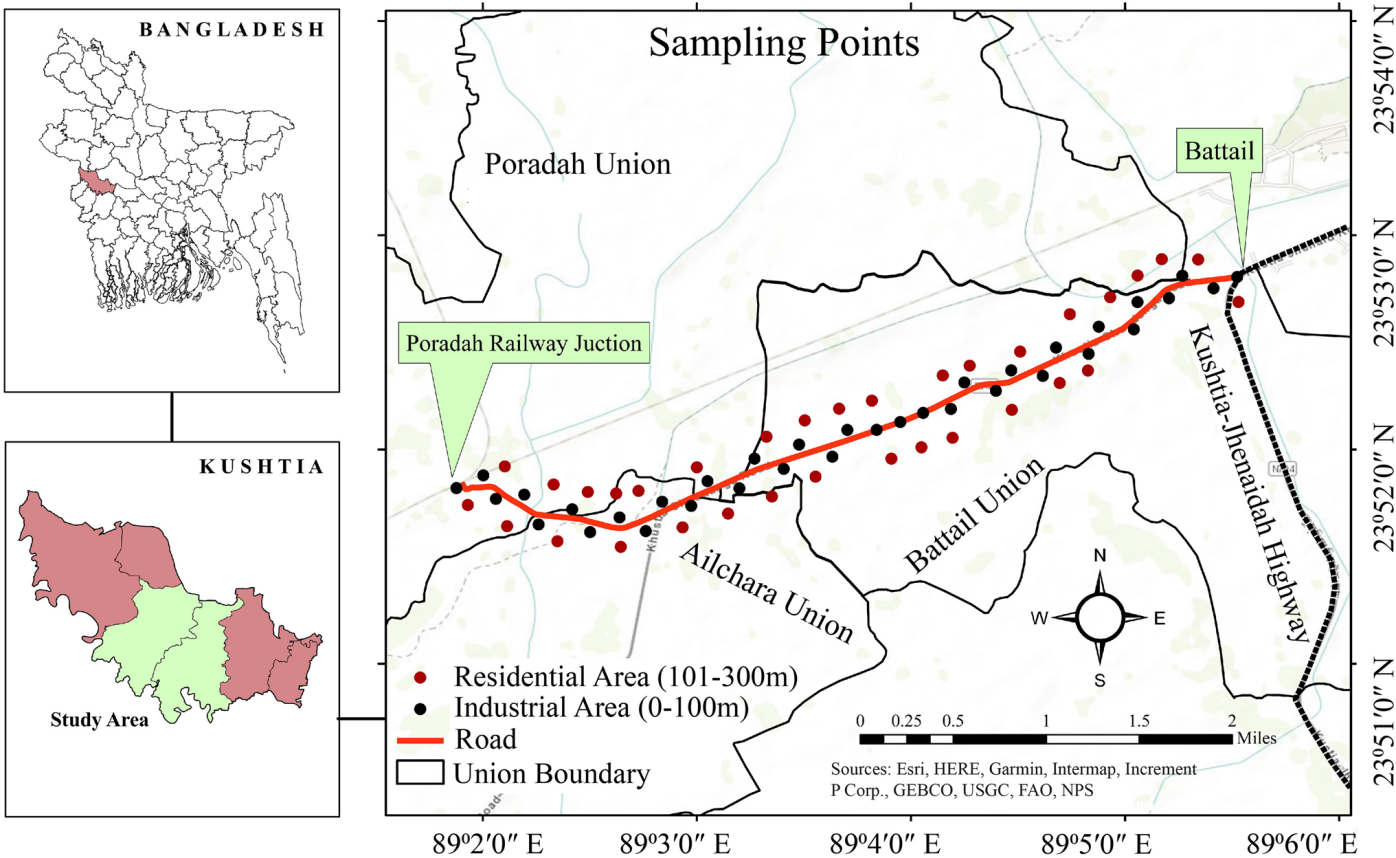
Study area, sampling points, and dispersion of both particulate matter concentration (PM_2.5_ and PM_10_) from the source points

This study focuses on the assessment of air particulate matter (APM) pollution from rice mills and its associated health risks in the Kushtia District, Bangladesh (Fig. 1). This study area was chosen to assess the impact of rice mill emissions on air quality in both industrial and nearby residential areas, providing insights into the ranges of pollutant dispersion and associated health risks. The absolute location of the study area is 23.74°N and 89.76°E, and it spans approximately 6 km from the Kushtia-Jhenaidah Highway to the Poradah railway station in Kushtia. Airborne particulate matter (PM), temperature, and relative humidity were measured simultaneously using an Air Particle Counter Meter (Model HT-9600) and a PM air-quality-optimised sensor (PMS-5003). Measurements were conducted at ~ 2.5 m above ground level, representing the human breathing zone, at both roadside and industrial micro-environments near rice processing hubs.

PM concentrations were collected from two distinct zones: 35 samples from industrial and roadside areas (mixed sources) located within 0–100 m of rice mills, and 34 samples from residential areas situated 101–300 m away from the mills. To ensure temporal consistency, measurements at each sampling location were taken twice daily (08:00–10:00 AM and 2:00–4:00 PM) over a continuous four-week period in December 2025 (Table S1), chosen for its stable winter atmospheric conditions, associated with lower boundary-layer heights, temperature inversions, reduced wind speeds, and limited atmospheric mixing, all of which favor pollutant accumulation and elevated PM concentrations, representing the period of greatest environmental and health concern in Bangladesh, when vulnerable populations are expected to experience the highest exposure burden. Therefore, the dataset provides a scientifically meaningful assessment of a “worst-case winter scenario” rather than an annualized exposure estimate.

For each sampling point, the average of the two daily measurements was used to represent the site’s PM concentration. Before data recording, each sensor was allowed a stabilisation period of approximately 5–7 min to reach local environmental equilibrium, during which temperature and relative humidity typically varied by less than 1–2 °C and 3–5%, respectively. Each sampling point’s local surroundings, including proximity to major roads, rice mills, and commercial areas, were documented to account for micro-environmental influences and minimise site-specific biases. Secondary meteorological data, including daily average wind speed and direction at 10 m height for each sampling coordinate, were retrieved from NASA’s POWER Data Access Viewer (https://power.larc.nasa.gov/data-access-viewer/), which provides satellite-derived data calibrated against ground stations (Table S2). This allowed assessment of meteorological influences on PM dispersion. By sampling multiple times per day at each site and documenting micro-environmental conditions, this approach ensures that all sampling points are effectively monitored with temporal and spatial consistency, providing a reliable baseline for estimating chronic exposure impacts of airborne PM near rice processing hubs.

### Device optimisation and data validation

Measuring PM concentration has become increasingly important, leading to the widespread use of low-cost, portable monitoring devices. Various companies now offer compact particulate matter sensors and mobile devices for assessing air quality in indoor and outdoor environments (Molaie and Lino 2021). In this study, we used the Air Particle Counter Meter (Sisco India HT-9600) to measure PM levels in both industrial and residential areas of Kushtia. This device demonstrates a PM measurement accuracy of ± 5%, operates within a temperature range of 0–50 °C (32–122°F), and measures relative humidity from 0 to 100% RH with an accuracy of ± 2% RH (Device Specifications: Sisco India HT-9600 Particle Counter). Similar devices have been used in previous studies; for instance, Bolla et al. (2021) applied the HT-9600 for spatial analysis of PM_10_ in limestone mining villages, and Mahmud et al. (2024) used it to study PM_2.5_ and PM_10_ dynamics in Rajshahi City; and most recently Hossain et al. (2026) utilized this devices to record the particulate matter and analyzed health risks in the industrial hubs in Bangladesh.

To ensure the reliability of the HT-9600 measurements, we used the HS-5003 particle counter sensor (PMS5003 High Accuracy Particle Concentration Sensor, Plan Tower) as a validation instrument. Before deployment, the HS-5003 was factory-calibrated using its integrated laser scattering reference module. At each sampling site, both the HT-9600 and HS-5003 sensors were co-located to measure PM concentrations simultaneously. A 5–7-min stabilisation period was maintained at each site to allow the sensors to equilibrate with ambient environmental conditions, reducing transient measurement noise. The readings from the two devices were then compared using linear regression analysis, which showed a strong correlation (R2 ≥ 0.95 (Fig. S1) for both PM_2.5_ and PM_10_), indicating ≥ 90% agreement. The average PM concentrations from both devices were subsequently used for further analysis, including health risk assessment (Table S2). This approach ensures robust validation of the low-cost HT-9600 sensor against the high-accuracy HS-5003 reference data. Finally, we acknowledge that sensor measurements may carry some uncertainty, especially in low-cost or portable devices; however, co-location and cross-validation with reference sensors in this study demonstrated ≥ 90% agreement, providing confidence in the reported PM concentrations.

### Spatial interpolation and geospatial analysis

Spatial interpolation is an essential technique in geospatial analysis for estimating continuous environmental variables and ascertaining values at unsampled sites (Yang et al. 2020). Inverse Distance Weighting (IDW) interpolation is a widely used and appropriate method for geospatial mapping, employing a linearly weighted averaging of sample sites (Ikechukwu et al. 2017; Gao et al. 2023). IDW interpolation expressly assumes that objects in closer proximity are more similar than those that are farther apart (Hodam et al. 2017). IDW will employ the measured values surrounding the projected site to forecast its value (Hossain et al. 2023). This study employed IDW using ArcGIS 10.8.2 to visualise and analyse the spatial distribution pattern of particulate matter (PM) in industrial and residential areas.

### Short-range dispersion of airborne PM

This study evaluated the dispersion of airborne PM through wind influences using the pollution rose diagram technique for industrial and residential areas separately, followed by a similar study by Sharma et al. (2022). Other researchers utilised a similar pollution rose diagram to observe surface wind speed at Minangkabau International Airport and analysed wind roses using hierarchical clusters (Oktavia et al. 2023).

### Dispersion of airborne PM using the Gaussian plume model

We employed the Gaussian plume model, one of the most established and widely applied approaches for estimating or predicting concentrations to simulate the dispersion of gaseous pollutants and particulate matter from specific point sources (Salih et al. 2025; Sinha et al. 2025; Tyovenda et al. 2021) and to simulate the dispersion of pollutants downwind from point sources in a steady-state atmosphere and under constant emission rates (Turner 2020). The model assumes that the pollutant concentration in the atmosphere follows a normal (Gaussian) distribution both vertically and horizontally.

In this study, we calculated the only horizontal dispersion distance from the source points based on the concentration threshold at which pollutant levels became negligible (below 0.1 µg/m^3^). High-level computer language-Python 3.8, leveraging libraries such as NumPy and SciPy for mathematical operations, and Pandas for data handling, and data visualisation was performed using Matplotlib and Origin Pro. The general form of the Gaussian plume equation is as follows:

1 $$C\left(x,y,z\right)=\frac{\mathrm{Q}}{2\pi \mu {\sigma}_{y}{\sigma}_{z}}\mathrm{exp}\left(-\frac{{y}^{3}}{{2{\sigma}_{y}}^{2}}\right)[\mathrm{exp}{\left(-\frac{z-{H}^{2}}{{{2\sigma }_{z}}^{2}}\right)}^{2}+\mathrm{exp}{\left(-\frac{z+{H}^{2}}{{{2\sigma }_{z}}^{2}}\right)}^{2}$$

C (x, y, z) denotes the pollutant concentration at a certain spatial position (µg/m^3^), Q represents the pollutant emission rate (g/s), µ indicates the wind speed (m/s), H signifies the height of the emission source (m), while σ_y_ and σ_z_ are the horizontal and vertical dispersion parameters (m). The pollutant emission rates considered for PM_2.5_ and PM_10_ were determined to be 1.4 × 10⁻^5^ g/s and 1.93 × 10⁻^5^ g/s, respectively. The wind speed was recorded at 2.54 m/s, and the height of the emission source was approximately 27 m (based on expert opinion). The atmospheric stability class was determined using previously established categories (Table S5) outlined by the U.S. Environmental Protection Agency (2020) and Mitchell and Timbre (1979). For this study, stability class B was selected, representing unstable atmospheric conditions typically associated with clear, sunny days and light to moderate winds during the winter season. Model validation was performed using a train-test split, with 70% of the dataset used for training and 30% for testing. Separate linear regression models were developed for the prediction of PM_2.5_ and PM_10_ dispersion. The models were evaluated using standard performance metrics, including R-squared (R^2^), Mean Absolute Error (MAE), Mean Squared Error (MSE), and Root Mean Squared Error (RMSE). These metrics were calculated from both the training and test datasets to assess model accuracy and generalisation. We recognise that various sources, such as open waste burning, biomass burning, vehicle emissions, and construction dust, can contribute to ambient PM concentrations. Emissions from rice processing mills are particularly represented as point sources in this study using the Gaussian Plume Model. The model mostly accounts for the influence of industrial sources, while some background PM may affect observed amounts at residential sites.

### Hazard quotient and non-cancer health risks

A human health risk assessment was conducted by estimating the hazard quotient (HQ). The HQ is an important statistic for assessing atmospheric PM and related human health hazards in any impact location. Exposure to humans was described using the average daily dosage (ADD), calculated using Eq. 2 (Amnuaylojaroen and Parasin 2023).

2 $$\mathrm{A}\mathrm{D}\mathrm{D}=\frac{\left(\mathrm{C} \times \mathrm{I}\mathrm{R} \times \mathrm{E}\mathrm{T}\times \mathrm{E}\mathrm{F} \times \mathrm{E}\mathrm{D}\right)}{\left(\mathrm{B}\mathrm{W} \times \mathrm{A}\mathrm{T}\right)}$$

where, IR refers the inhalation rate (IR) = 0.83 m^3^/h, pollutant concentration (C) (µg/m^3^), exposure frequency (EF) = 350 days/year, exposure duration (ED) = 30 years, exposure time (ET) = 24 h/day, averaging time (AT) = ED × 365 days/year, and body weight (BW) = 70 kg. In this context, IR signifies the inhalation rate (m^3^/day), C represents the pollutant concentration (µg/m^3^), EF denotes the exposure frequency (days/year), ED indicates the exposure duration (years), ET refers to the exposure time (hours/day), AT stands for the average exposure time (days), and BW represents body weight (kg).

3 $$\mathrm{R}\mathrm{f}\mathrm{d}=\frac{\left(\mathrm{R}\mathrm{f}\mathrm{c} \times \mathrm{I}\mathrm{R} \times \mathrm{E}\mathrm{T}\times \mathrm{E}\mathrm{F} \times \mathrm{E}\mathrm{D}\right)}{\left(\mathrm{B}\mathrm{W} \times \mathrm{A}\mathrm{T}\right)}$$

4 $$\mathrm{H}\mathrm{Q}=\frac{ADD}{Rfd}$$

The non-carcinogenic health risk assessment was performed by calculating the Hazard Quotient (HQ) using Eq. (4), following the approach described by Algarni et al. (2021). In this method, the average daily dose (ADD) and the inhalation reference dose (R_f_d) were key parameters. The inhalation reference dose (R_f_d) was derived from Eq. (3) as proposed by Amnuaylojaroen and Parasin (2023). The inhalation reference concentration (R_f_c), representing the safe exposure threshold established by the United States Environmental Protection Agency (USEPA) under the National Ambient Air Quality Standards (US EPA, 2010), is 35 µg/m^3^ for PM₂.₅ and 150 µg/m^3^ for PM₁₀. When the HQ value is equal to or below unity (HQ ≤ 1), the likelihood of adverse health effects is considered negligible. Conversely, an HQ value greater than one (HQ > 1) indicates an increased probability of non-carcinogenic health risks, suggesting potential adverse effects from exposure.

### AirQ + tool-based health risk assessment

The AirQ + tool is a suitable tool that enables users to assess the potential impacts of air pollution exposure on human health risks, including the possibility of specific diseases, over a given period (Omidi et al. 2016; Yari et al. 2016). The AirQ^+^ proposed by the World Health Organization (WHO), was utilized to assess mortality and morbidity at the city scale (Javanmardi et al. 2018). Furthermore, AirQ + determines the ratio of cases associated with specific air pollutant concentrations, the incidence of cases per 100,000 individuals in the at-risk population, and the distribution of cases across various air pollutant concentration categories. It accomplishes this by employing health research reference rates, a threshold value, and relative risk metrics. AirQ + assesses the effects of PM exposure by an attributable percentage (AP) function, as delineated in Eq. 5.

5 $$\mathrm{A}\mathrm{P}=\frac{\left(\sum (RR-1) \;\times\;\;\mathrm{P}\right)}{\sum RR\;\times\;P}$$

AP denotes the proportion of health impacts within a designated population that may be ascribed to exposure to an air pollutant. This presumes a robust correlation between the exposure and the health concern, devoid of substantial variables affecting this association (Arregocés et al. 2023), where ‘RR’ denotes the relative risk for the health outcome in a population exposed to PM-contaminated air, and ‘P’ represents the percentage of the population that is exposed. Table 1 presents the relative risk (RR) estimates utilised in this analysis, derived from systematic reviews and research investigating the impact of prolonged particulate matter (PM) exposure on overall and cause-specific mortality, as documented by Chen and Hoek (2020) and Burnett et al. (2018). The research used standard relative risk values for specific diseases. These values were established by the WHO and are integral to the AirQ + model. The quantity of health problems linked to PM_10_ exposure (IE) and the number of instances associated with exposure (NE) can be calculated using Eqs. 6 and 7, respectively (Arregocés et al. 2023).

6 $$\mathrm{I}\mathrm{E}=\mathrm{I}\times \mathrm{A}\mathrm{P}$$

7 $$\mathrm{N}\mathrm{E}=\mathrm{I}\mathrm{E}\times \mathrm{N}$$

where I is the baseline incidence rate, and N is the total population exposed to the pollutant.

**Table 1:** The concentration of particulate matter in rice processing industrial and residential areas is a concern in this study and other countries globally

	Country/Places	(Average ± SD) (µg/m^3^)		References
		PM_2.5_	PM_10_	
International	Beijing, China	47.6 ± 42.0	75.0 ± 56.7	Hu et al. (2022)
	Bengaluru, India	32.13 ± 18.87	95.61 ± 42.55	Sowmya et al. (2024)
	Karachi, Pakistan	55.99	105.00	Malhi et al. (2023)
	Brazil	10.6 ± 7.2	25 ± 11	Cruz et al. (2024)
	New York	25.4 ± 47.1	25.4 ± 47.1	Lu et al. (2024)
	South Africa	106.38 ± 55.36	189.95 ± 103.54	Barhadje et al. (2024)
National	Dhaka	32.7 ± 20.3	NA	Rahman et al. (2021)
	Dhaka	44.1	91.7	Hoque et al. (2020)
	Industrial Area, Kushtia	93.26 ± 14.007	128.35 ± 21.61	This Study
	Residential Area, Kushtia	114.43 ± 15.56	154.36 ± 19.72	This Study
	BD Standards (24 h)	65	150	Hossain et al. (2026)
	WHO Guideline (24 h)	25	50	Hossain et al. (2026)
	EPA	35	150	U.S. EPA (2012)

*BD* Bangladesh, *WHO* World Health Organization, *NA* Not Availa

To quantify the long-term effects of PM_2.5_ and PM_10_, we used the baseline data provided in Table 1 and the average PM concentration for two areas respectively, a cut-off value of annual PM concentrations of 15 µg/m^3^ for PM_10_ and 2.4 µg/m^3^ for PM_2.5_ as recommended by the WHO (2021a, b) to quantify the health risk due to the exposure of PM concentrations. The data regarding the population at risk in the industrial and residential areas and the mortality data of Bangladesh were utilised (https://www.who.int/data/gho: WHO 2019). According to Hossain et al. (2026),  the main methodologies use evidence from epidemiological cohort studies showing a relationship between average short-term and long-term air pollution concentration levels and mortality risks in exposed populations. Health effects were assessed using GBD 2020 (Integrated Function 2019) for the following conditions: lung cancer mortality (ages 30 +), mortality from acute lower respiratory infections (ages 0–5), chronic obstructive pulmonary disease (COPD) mortality (ages 30 +), ischemic heart disease (IHD) mortality (ages 30 +), and stroke mortality (ages 30 +), as shown in Table S7.

### Statistical analysis

All statistical analyses were conducted in Origin Pro (v-2025) and JMP Pro (v-26). First, the Shapiro–Wilk test showed that the parameters are not normally distributed. Therefore, the non-parametric Kruskal–Wallis test, also known as non-parametric analysis of variance (ANOVA) was run. Then, a post hoc pairwise comparison was conducted using Dunn's and Conover's tests to evaluate variations in PM2.5 and PM10 across industrial and residential areas. Secondly, linear regression was employed to observe the relationship between particulate matter and temperature and relative humidity.

## Results

### Particulate matter (PM) concentration in the area

In the industrial area, the average concentration of PM_2.5_ was 93.26 µg/m^3^, ranging from 71.63 to 139.38 µg/m^3^, and the average concentration of PM_10_ was 128.35 µg/m^3^, ranging from 98.63 to 194.50 µg/m^3^ (Table 2; Table S3). In residential areas, the average concentration of PM_2.5_ was 114.43 µg/m^3^, ranging from 83.50 to 165.38 µg/m^3^, and the average concentration of PM_10_ was 154.36 µg/m^3^, ranging from 115.25 to 223.38 µg/m^3^ (Table S3). In industrial areas, the average PM_2.5_ concentration exceeds the BD, WHO, and EPA standards, but the average PM_10_ concentration exceeds the WHO standard only.

**Table 2:** Post-hoc analysis (Kruskal’s Wallis Test) of particulate matter (PM_2.5_, PM_10_) between industrial area and the residential area

Dunn's Test					
*Parameters*	*Sampling Places*	*Mean Rank Differences*	*Z values*	*p-Values*	*Significance*
PM_2.5_	Industry- Residence	−24.40	−5.08	3.64E-7	1
PM_10_		−23.99	−5.0004	5.72E-7	1
Relative Humidity		−2.76	−0.57	0.56	0
Temperature		15.51	3.23	0.001	1
Wind Speed		−3.97	−0.83	0.41	0
Conover's Test					
*Parameters*	*Sampling Places*	*Mean Rank Differences*	*Z values*	*p-Values*	*Sig*
PM_2.5_	Industry- Residence	−24.40	−6.44	1.56E-8	1
PM_10_		−23.99	−6.27	3.16E-8	1
Relative Humidity		−2.76	−0.57	0.57	0
Temperature		15.51	3.49	8.56E-4	1
Wind Speed		−3.97	−0.83	0.41	0

Significance equals 1, which indicates that the difference in the means is significant at the 0.05 level

Sigificance equals 0, which indicates that the difference in the means is NOT significant at the 0.05 level

**Fig. 2:**
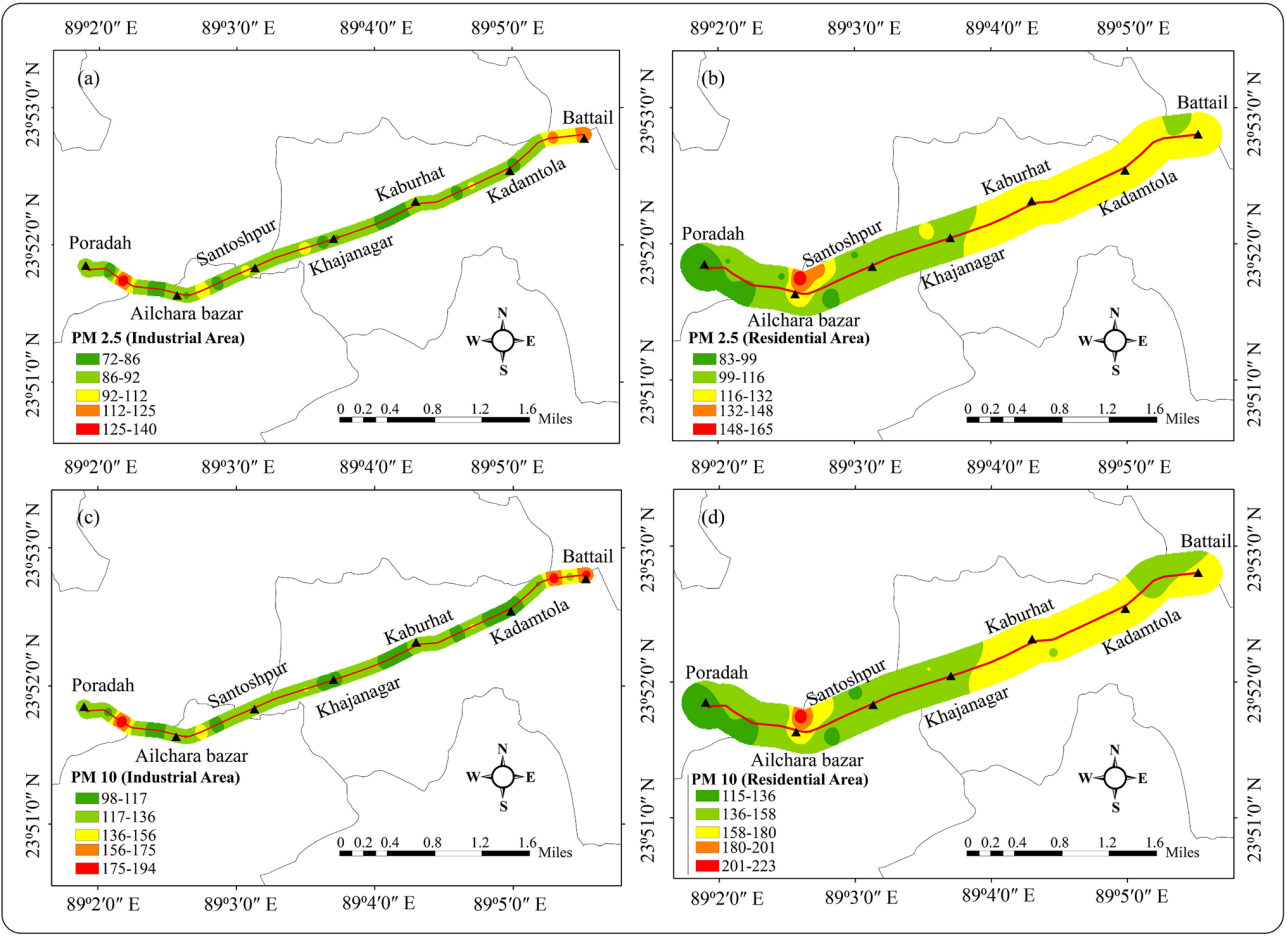
Spatial pattern and variation of particulate matter concentration (**a**) PM_2.5_ and (**b**) PM_10_ in the industrial area, and (**c**) PM_2.5_ and (**d**) PM_10_ in the residential area, respectively

From (Fig. 2a-b), the highest concentration of PM_2.5_ and PM_10_ is found in the westernmost and easternmost parts, and the lowest in the central part of the industrial area. The concentration of PM_2.5_ exceeds all BD, WHO, and EPA standards throughout the area; meanwhile, PM_10_ concentrations exceed all standards at the easternmost and westernmost parts, and only the WHO standards at the central part. From Fig. 2c-d in the residential area, the highest PM_2.5_ and PM_10_ concentration is found in the western part. Moreover, most of the eastern part showed higher concentrations of both PM_2.5_ and PM_10_. Similar to industrial areas, PM_2.5_ concentrations exceed all BD, WHO, and EPA standards, and PM_10_ concentrations exceed these limits throughout the residential area, except at the westernmost part.

### Variation in PM concentration

The results of the Kruskal–Wallis test using Dunn’s post hoc analysis revealed there is a significant difference in PM_2.5_ (Mean rank Differences = −24.40, *p* > 0.05) and PM_10_ (Mean rank Differences = −23.99, *p* > 0.05) between the industrial and residential areas. Air temperature also showed a significant difference between these two areas (Mean rank Difference = 15.51, *p* > 0.05). The results also demonstrated that there is no significant difference in relative humidity (Mean rank Differences −2.76, *p* > 0.05) and wind speed (Mean rank Differences −3.97, *p* > 0.05) between industrial areas and residential areas. These findings are also supported by Conover’s test (Table 3).

**Table 3:** Potential non-carcinogenic health risk assessment of observed particulate matter concentrations of the industrial area and the residential area

Sampling Location	Total sample	Hazard Quotient (HQ)			
		PM_2.5_		PM_10_	
		High Risk/Range (HQ > 1)	Low Risk/Range (HQ < 1)	High Risk/Range (HQ > 1)	Low Risk/Range (HQ < 1)
Industrial Area, Kushtia	*n* = 35	(2.04–3.98) (*n* = 35)	–	(1.19–1.29) (n = 3)	(0.66–0.99) (*n* = 32)
Residential Area, Kushtia	*n* = 34	(2.38–4.72) (*n* = 34)	–	(1.005–1.48) (*n* = 18)	(0.76–0.99) (*n* = 16)
Overall Study Area	*n* = 69	(2.04–4.72) (*n* = 69)	–	(1.005–1.48) (*n* = 21)	(0.66–0.99) (*n* = 48)

### Effects of meteorology on PM concentration

**Fig. 3:**
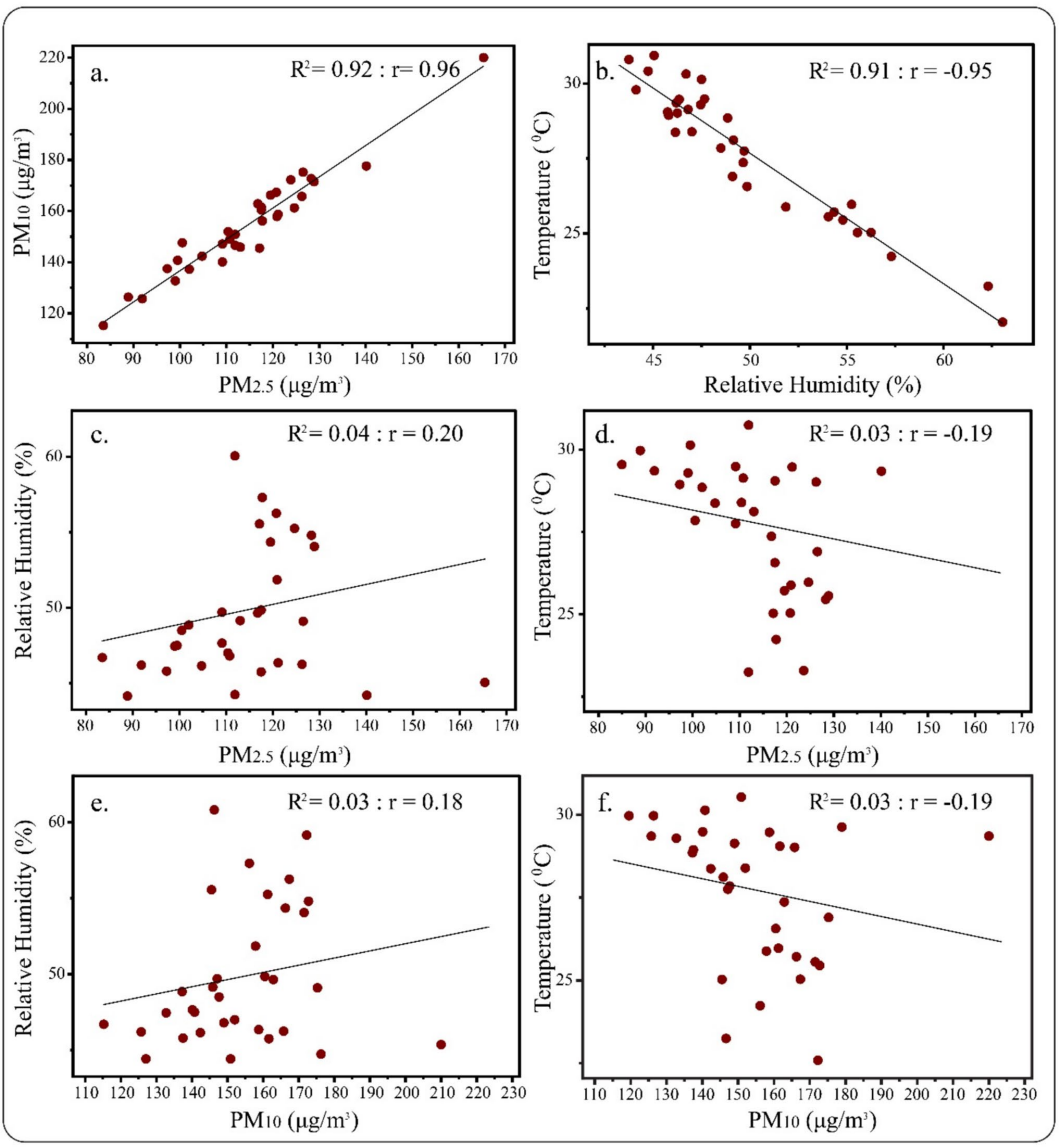
Linear Regression analysis among particulate matter and meteorological parameters in the sampling areas. The findings indicated that both PMs are strongly and positively associated with each other, while temperature and humidity showed an inverse pattern. However, both PMs with relative humidity showed slightly positive correlations, and PMs with temperature showed negative correlations, respectively

Figure 3 illustrates the linear correlation between particulate matter concentrations and meteorological parameters. The results revealed that there was a strong positive (R^2^ = 0.92) correlation between PM_2.5_ and PM_10_ (Fig. 3a). Figure 3b represents the relationship between temperature and relative humidity, and the result indicates there was also a strong negative association between them (R^2^ = 0.91 and r = −95). Both PM_2.5_ and PM_10_ show that there is a weak positive correlation (r = 0.20, r = 0.18, respectively) with relative humidity (Fig. 3c and e). On the contrary, temperature showed a weak negative correlation (r = −0.19) with PM_2.5_ and PM_10_, as shown in Fig. 3d and f.

### Dispersion of particulate matter (PM)

**Fig. 4:**
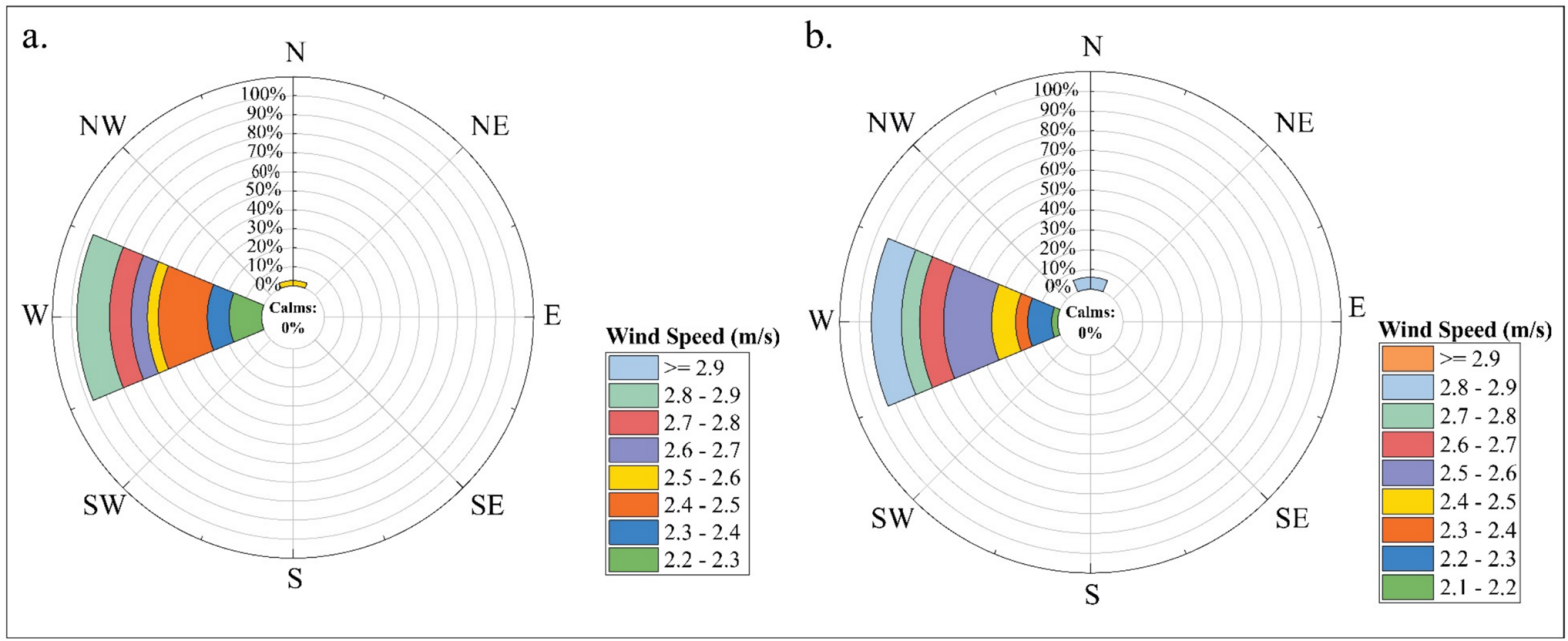
Speed and direction of wind velocity (m/s)- **(a)** in the industrial area and **(b)** in the residential area

Figure 4 represents the wind speed as well as the direction dispersion of the study area during the study period. The two wind rose diagrams illustrate the frequency and intensity of wind directions and wind speeds in the study area, with wind directions indicating the origin from which the wind is blowing (Fig. 4a and b). Both diagrams reveal a dominant westerly wind direction, indicating that winds predominantly blow from west to east, accounting for nearly 100% of the wind. The results indicated that the wind speed ranged from 2.1 m/s to > 2.9 m/s for both industrial and residential areas. The results also revealed that wind speed dispersed from the Eastern part of the study area (Battail and surrounding area) to the Western part, including Khajanagr and Ailchara Bazar.

**Fig. 5:**
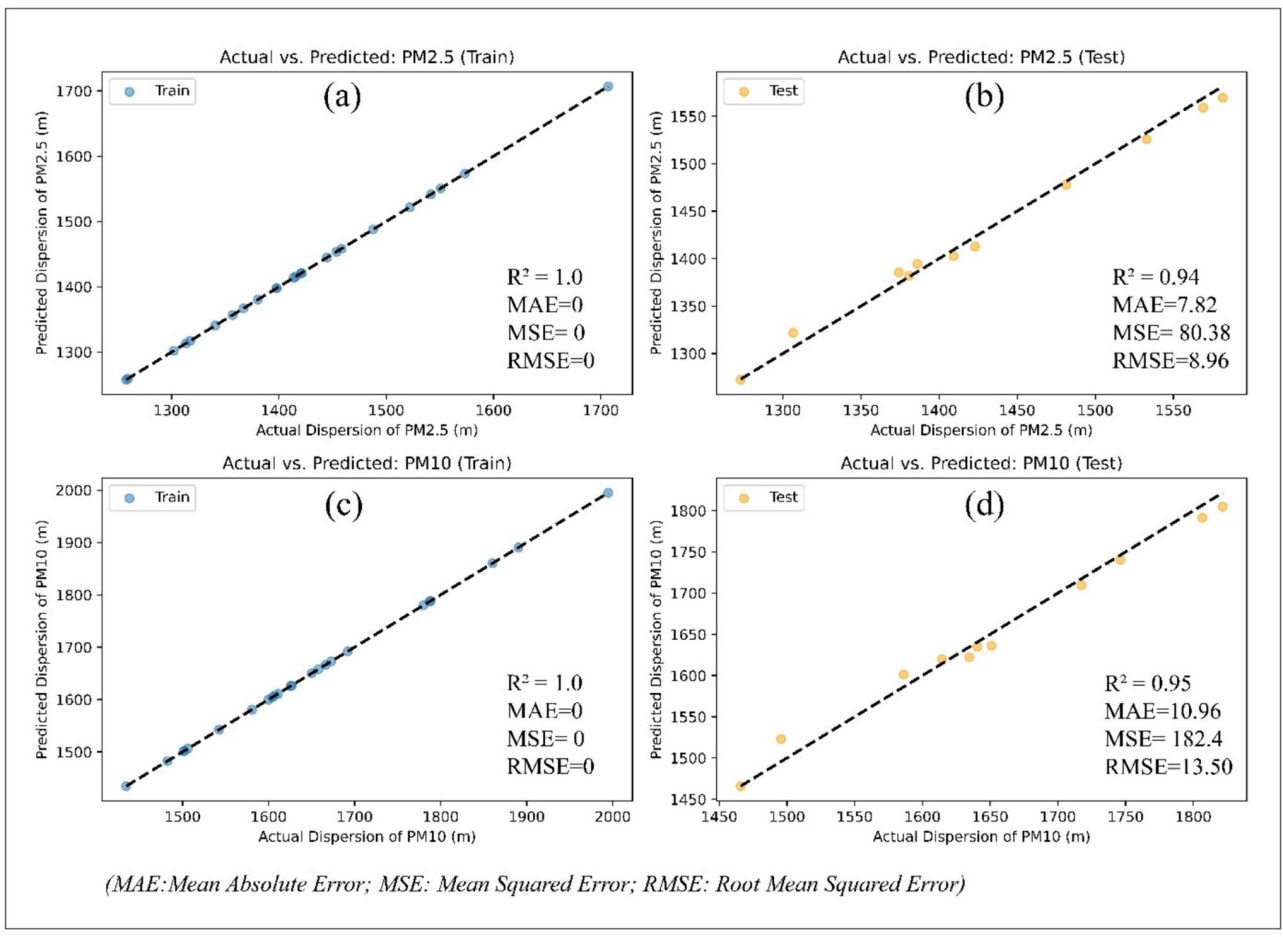
Gaussian Dispersion Model validation plots (**a**) training (70% data) and (**b**) testing data (30% data) for PM_2.5,_ while (**c**) training (70% data) and (**d**) testing data (30% data) for PM_10_ dispersion distances show strong agreement between actual and predicted values with high R^2^ and low error metrics for training and testing datasets

To evaluate the dispersion nature of both PM, Fig. 5. illustrates the high-level language-based Gaussian Dispersion Model validation plots. Fig. 5a represents 70% training data, Fig. 5b represents 30% testing data for PM_2.5_, while Fig. 5c represents training data of 70%, and Fig. 5d represents testing data of 30% for PM_10_. Dispersion distances show strong agreement between actual and predicted values, with high R^2^ and low error metrics across the training and test datasets.

**Fig. 6:**
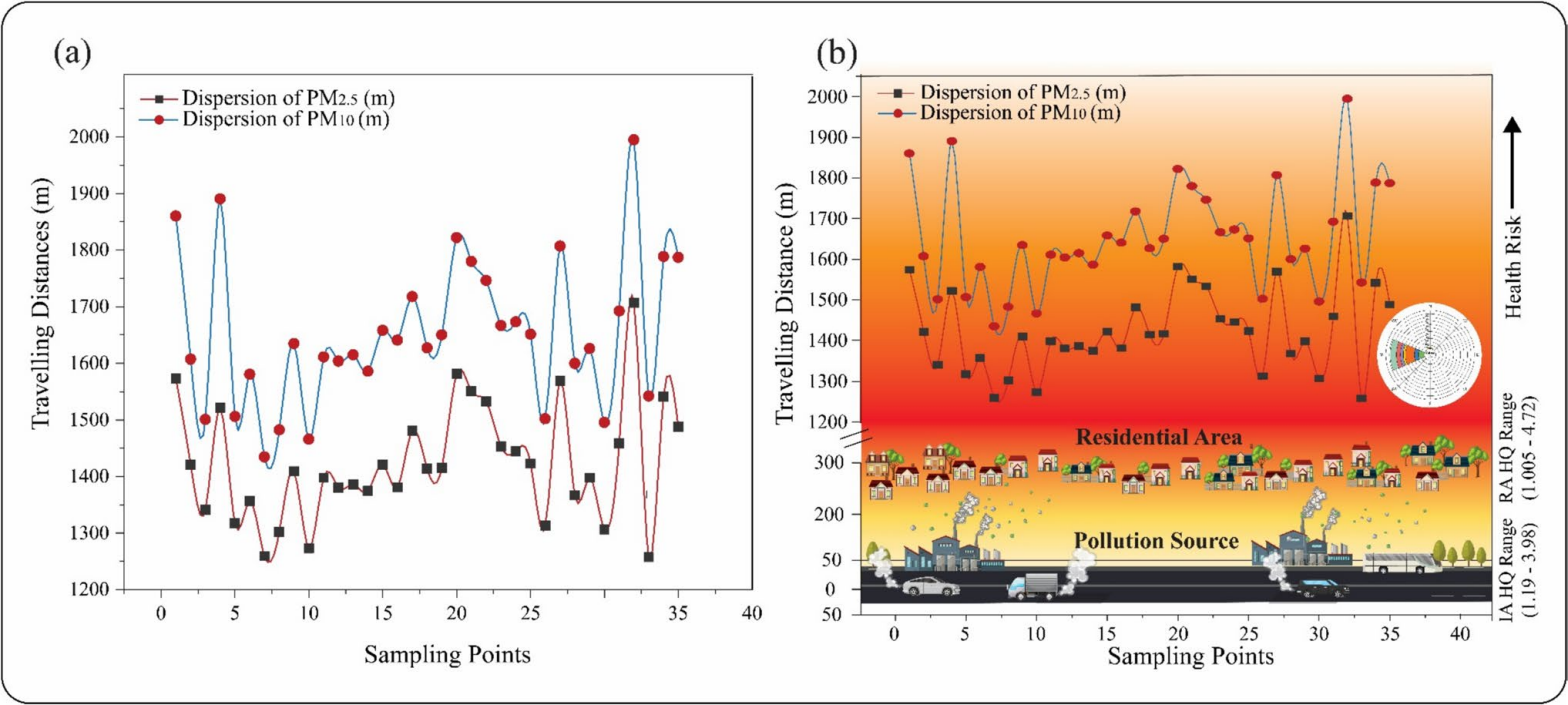
Gaussian Dispersion Modeling based evaluation (**a**) Travelling distances of PM_2.5_ and PM_10_ from mixed source sampling points, indicating higher dispersion potential of PM_10_ compared to PM_2.5_. Travelling ranges of PM_10_ cover up to 1994.8 m from the source point, while PM_2.5_ travels up to 1706.9 m. The residential area covers up to 300 m, considering source points (0–50 m); (**b**) Health risk associated with PM contamination and the possible range of areas covered by the effects. A residential area showed high vulnerability due to the high concentrations of PMs and traveling approach with the wind direction from the source points

Figure 6a illustrates the travelling distances of particulate matter (PM_2.5_ and PM_10_) from various mixed-source locations, characterised by the coexistence of rice processing industries and heavy vehicular traffic, along roadside sampling points. The results clearly show that PM_10_ consistently travels farther than PM_2.5_ across most sampling points. The highest dispersion distance for PM_10_ is observed at sampling point 32, reaching 1994.8 m, indicating a strong emission source or favourable dispersion conditions, such as wind turbulence or thermal uplift. In contrast, PM_2.5_ reaches its maximum travelling distance at the same point (32), but only about 1706.9 m, highlighting a notable difference in dispersion capability between the two particle sizes. On the lower end, PM_2.5_ shows the lowest travelling distance at sampling point 33, measuring just about 1257.7 m, suggesting a weak emission source or unfavourable dispersion conditions at that location. For PM_10_, the shortest travelling distance is observed around sampling point 7, at approximately 1434.5 m, which is still significantly higher than the minimum value for PM_2.5_. The dispersion of PM_10_ is wider than that of PM_2.5_, which is probably because of the mechanical effects of vehicles and industries (Table S6). The spatial peaks and troughs are indicative of changes in emissions intensity and local environmental conditions.

It shows that the minimum distance the particulate matter travelled from the source points is 1257.7 m, indicating that PM particles have already reached the residential areas, which are at distances of 101–300 m from the source. In Fig. 6b, the health risks associated with PM emissions are visualised through the Hazard Quotients (HQ), with higher HQ values indicating greater risks to human health. The colour gradient in the figure highlights these varying risks, with the industrial area (0–100 m from the pollution source) showing a relatively higher risk range (HQ: 1.9–3.98). However, as the PMs travel from the source to the surrounding residential areas, the dispersion distances increase significantly. This is critical, as HQ values in residential areas have been reported as high as 4.72, suggesting that the health risks in these regions are not only significant but potentially severe. The dispersion of PMs over such long distances clearly demonstrates that residential areas, even at considerable distances from the industrial zone, are already experiencing significant health risks from ongoing exposure to particulate matter.

### Non-carcinogenic health risks

Non-carcinogenic health risks were estimated using the Hazard Quotient (HQ), and it was observed that effects vary across sampling locations with respect to individual concentrations (Table S4). The results showed that due to PM_2.5_ in industrial areas_,_ all the sampling points (*n* = 35) showed potential for high health risks (HQ = 2.04–3.98), and for PM_10,_ 3 out of 35 sampling points showed potential for high health risks (HQ = 1.19–1.29) and 32 out of 35 sampling points showed potential for low health risks (HQ = 0.66–0.99). In contrast, in the residential area, due to PM_2.5,_ all sampling points (*n* = 33) were at high health risks (HQ = 2.38–4.72), and 18 sampling points were at high risks (HQ = 1.005–1.48), and 15 sampling points exhibited low health risks (HQ = 0.76–0.99) due to PM_10_ concentration (Table 4). The results demonstrated that PM_2.5_ at all sampling points exceeds the HQ limit and poses a very high health risk in both industrial and residential areas.

**Table 4:** Baseline incidence (BI) and relative risk for this study with a 95% confidence interval (95% CI)

RR values (95% CI) per 10 µg/m^3^				
Long-term Diseases	NI	BI	PM_2.5_	PM_10_
ALRI	2702	1.65	1.007 (1.0029–1.01)	–
COPD	10,266	6.29	–	–
IHD	28,469	17.46	1.006 (1.002–1.02)	1.06(1.01–1.1)
Stroke	25,651	15.73	1.009 (1.001–1.01)	–
LC	2133	1.30	1.009 (1.0039–1.01)	1.08(1.04 −1.13)

*ALRI* Acute Lower Respiratory Infection for Children, *COPD* Chronic obstructive pulmonary disease for Adults, *IHD* Ischemic Heart Disease for Adults, *LC* Lung Cancer for Adults, *BI* Baseline Incidence (Crude rate per 100,000 inhabitants); The values in parentheses represent the low and high relative risks; *NI* Total Number of Incidents, *RR*  Relative Risks (CI: 95%) per 10 µg/m^3^

### Air Q + tools-based health risks

Table 5 presents the estimated attributable proportion (%), the estimated number of attributable cases, and the estimated number of attributable cases per 100,000 at-risk population for some major diseases in children (< 5 years) and adults (30 + years) working and living in polluted areas due to PM contamination.

**Table 5:** Potential health risk analysis using AirQ + for different age groups due to various diseases of the inhabitants living in the impacted industrial area and residential areas

Sampling Locations	Air particulate matter (APM)						
	PM_2.5_				PM_10_		
	DN	EAP	EAC	EACP	EAP	EAC	EACP
Residential Area, Kushtia	ALRI	39.78 (29.83–48.99)	0	0.66 (0.49–0.81)	–	–	–
	COPD	48.01(40.95–55.3)	2 (1–2)	3.02 (2.58–3.48)	–	–	–
	IHD	56.25 (45.59–64.6)	6 (5–7)	9.82 (7.96–11.28)	55.6 (12.95–73.51)	6(1–7)	9.71 (2.26–12.83)
	Stroke	65.05 (55.01–72.45)	6 (5–7)	10.23 (8.65–11.40)	–	–	–
	LC	34.16 (25.31–41.83)	0	0.44 (0.33–0.54)	65.79 (42.11–81.79)	0–1	0.86 (0.55–1.06)
Industrial Area, Kushtia	ALRI	–	–	–	–	–	–
	COPD	43.47 (36.26–50.72)	1 (0–1)	2.73 (2.28–3.19)	–	–	–
	IHD	56.01 (45.55–64.53)	2 (2–2)	9.78 (7.95–11.27)	48.34 (10.67–66.05)	2(0–2)	8.44 (1.86–11.53)
	Stroke	64.84 (54.57–72.15)	2 (2–2)	10.20 (8.58 −11.35)	–	–	–
	LC	33.03(24.77–40.49)	0	0.43 (0.32–0.53)	58.2 (35.89–74.98)	0	0.76 (0.47–0.97)
Overall Study Area	ALRI	39.78 (29.83–48.99)	0	0.66 (0.49–0.81)	–	–	–
	COPD	45.85 (38.72–53.12)	2 (2–3)	2.88 (2.44–3.34)			
	IHD	56.18 (45.56–64.55)	8 (6–9)	9.81 (7.95–11.27)	52.11 (11.81–70.01)	7 (2–9)	9.10 (2.06–12.2)
	Stroke	65 (54.86–72.33)	8 (7–9)	10.23 (8.63–11.38)	–	–	–
	LC	33.68 (25.12–41.29)	0	0.44 (0.33–0.54)	62.19 (39.08–78.65)	1 (0–1)	0.81 (0.51–1.02)

*ALRI* Acute Lower Respiratory Infection for Children, *COPD* Chronic Obstructive Pulmonary Diseases for Adults, *IHD* Ischemic Heart Diseases for Adults, *LC* Lung Cancer for Adults; Nill values mentioned the Not Applicable for PM_10_ to calculate estimated health risks of those specific diseases using AirQ + tools, *DN* Disease Name, *EAP* Estimated attributable proportion (%), *EAC* Estimated number of attributable cases, *EACP* Estimated number of attributable cases per 100,000 at-risk population

#### Acute lower respiratory infection (ALRI) for children

The estimated percentage of mortality attributed to prolonged exposure to PM_2.5_ among children (< 5 years) due to Acute Lower Respiratory Infections (ALRI) in residential areas is 39.78 (29.83–48.99), and the estimated number of attributable cases per 100,000 at-risk population was 0.66 (95% CI: 0.49–0.81). The corresponding estimate for attributable deaths among children was < 1 (effectively 0 after rounding), reflecting extremely small model-derived values within the population (Table 5).

#### Chronic obstructive pulmonary disease (COPD) for adults

Table 5 demonstrates the estimated proportion of death cases attributable to prolonged exposure to PM_2.5_ for Chronic obstructive pulmonary disease (COPD) in adults aged 30 + years. The proportions are 48.01 (40.95–55.3) and 43.47 (36.26–50.72) in residential and industrial areas, respectively, and the overall study area proportion is 45.85 (38.72–53.12). Also, the number of deaths attributed to COPD due to PM_2.5_ exposure was 2 (1–2) in residential areas and 1 (0–1) in industrial areas, respectively, and the number of deaths attributed to the overall study area was 2 (2–3). Moreover, the estimated number of attributable cases per 100,000 at-risk population is 3.02 (2.58–3.48) and 2.73 (2.28–3.19), respectively. On the contrary, the overall study area showed an estimated attributable case rate of 2.88 per 100,000 at-risk population (2.44–3.34).

#### Ischemic heart disease (IHD) for adults

The estimated percentage of mortality attributable to prolonged exposure to PM_2.5_ among individuals aged 30 years and above due to ischemic heart disease (IHD). The average mortality proportion is almost identical in residential and industrial areas, at 56.25 and 56.01, respectively, while the overall study area shows a value of 56.18. These estimates correspond to 6 (5–7) deaths in residential areas, 2 (2–2) deaths in industrial areas, and a total of 8 (6–9) deaths across the study area. The calculated mortality rates per 100,000 individuals at risk are 9.82 (7.96–11.28), 9.78 (7.95–11.27), and 9.81 (7.95–11.27) for residential, industrial, and overall areas, respectively. By comparison, long-term exposure to PM_10_ yields estimated IHD mortality percentages of 55.6 (12.95–73.51) in residential areas and 48.34 (10.67–66.05) in industrial areas, with an overall rate of 52.11 (11.81–70.01) for the study area. This corresponds to a maximum of 7 deaths in residential areas and 2 deaths in industrial areas. The related mortality rates range from 2.26–12.83 and 1.86–11.53 per 100,000 individuals at risk in residential and industrial areas, respectively (Table 5).

#### Stroke for adults

Table 5 expresses the estimated mortality percentages by stroke of adults aged 30 years and above, which are 65.05 (55.01–72.45) in residential areas and 64.84 (54.57–72.15) in industrial areas. The number of stroke-related deaths due to PM₂.₅ exposure is estimated to be up to 7 in residential areas and 2 in industrial areas. Correspondingly, the mortality rates per 100,000 at-risk population range from 8.65 to 11.40 in residential areas and from 8.58 to 11.35 in industrial areas.

#### Lung cancer (LC) for adults

The estimated mortality due to PM_2.5_ exposure for lung cancer among adults aged 30 + is reported as 25.31–41.83 in residential areas and 24.77–40.49 in industrial areas. However, 0 (zero) deaths in both areas. The corresponding mortality rates per 100,000 individuals at risk are very low, 0.44 (0.33–0.54) and 0.43 (0.32–0.53) for residential and industrial areas, respectively. By contrast, exposure to PM_10_ yields higher estimated mortality percentages for lung cancer of 65.79 in residential areas and 58.2 in industrial areas. These figures result in approximately 1 (one) death in residential areas and no deaths observed in industrial areas, with mortality rates of only 0.86 and 0.76 per 100,000 individuals at risk, respectively. Overall, the estimated number of attributable cases per 100,000 in the study area is 0.44 for PM₂.₅ and 0.81 for PM_10_ (Table 5).

## Discussion

### Pollution level assessment

The elevated levels of PM_2.5_ and PM_10_ in both industrial and residential zones of the study area indicate a significant air pollution problem. This pollution problem may pose severe public health risks among children and adults. In the industrial area, the average PM_2.5_ concentration was 93.26 µg/m^3^, while the average PM_10_ concentration was 128.35 µg/m^3^. These levels of PM concentration exceed the national air quality standard of Bangladesh (BD), the World Health Organisation (WHO), and the United States Environmental Protection Agency (EPA) guidelines. The higher concentration of PM in rice processing industrial areas may result from different sources, such as emissions from rice processing mills, vehicular traffic, construction activities, etc. (Begum et al. 2011). While in a residential area, the situation is way more concerning. The average concentration of PM_2.5_ (114.43 µg/m^3^) is significantly elevated compared to the industrial area, indicating the extensive level of air pollution, not only exacerbated by vehicular emissions, biomass combustion, and unregulated construction activities (Begum et al. 2010) but also the prevailing east–west wind patterns, dispersing the particulate matter. The spatial distribution of particulate matter indicates that the emissions generated by industrial and traffic-prone eastern areas are transported west to the residential areas where they accumulate. Similar findings are reported by Molina et al. (2010), who have stressed the need for wind corridors to define the transport and deposition of pollutants from source points. The high levels of PM_10_ (mean = 154.36 µg/m^3^) exceed WHO and national air quality standards, indicating that residents in such areas experience poor air quality, consistent with past evidence on exposure to PM and its health effects (World Health Organization (2021a, b). Another significant finding of the present study is that PM_10_ exceeded WHO limits in only a few industrial sectors, whereas PM_2.5_ exceeded all critical thresholds across most of the study areas, especially in residential zones. The results indicate that PM_2.5_ levels were significantly above the national and WHO limits in most zones, and the highest concentrations were observed in a residential area, demonstrating a severe air quality threat from industrial pollution. High PM_2.5_ levels in residential areas mainly result from the use of biomass fuels for cooking and heating, open burning of household and municipal waste, and heavy vehicle traffic, which generate exhaust emissions and resuspended dust (Weaver et al. 2019). These are diffused and incorporated in everyday activities, and there is endemic local exposure. Conversely, controlled fuel use, point-source control, and improved dispersion owing to spatial organisation may result in lower PM_2.5_ levels in some industrial zones. These trends are in line with previous studies of source apportionment in Bangladesh, which reported biomass burning, traffic emissions, brick kilns, and road dust to be the key contributors (Salam et al. 2003), and with other global results that household combustion is the primary contributor to outdoor fine particle pollution. The repeated exceedance of safety limits, especially in residential areas, underscores the urgent need to implement strict pollution monitoring and mitigation measures in urban Bangladesh. Global studies shows evidence that particulate matter pollution exhibits strong spatial variability across both developed and developing regions. In Africa, elevated PM₂.₅ levels have been linked to biomass burning, waste combustion, and inefficient energy use, resulting in widespread exposure across urban and rural environments (Gahungu et al. 2022; Gordon et al. 2023). In Europe, source apportionment studies show that industrial activities, agriculture, transport, and residential combustion collectively contribute to PM₂.₅ pollution with strong land-use driven spatial heterogeneity (Zauli-Sajani et al. 2024). Biomass combustion also remains a notable contributor in several European regions, particularly in rural areas (Steiner and Lanzerstorfer 2024). These findings demonstrate that agro-industrial and combustion-based particulate emissions are a global issue.

### Impact of meteorological parameters on particulate matter

Meteorological parameters and their effects on ambient PM concentrations are important in the dynamics of air quality. The results of this study show a strong linear relationship between PM_2.5_ and PM_10_ concentrations (R2 = 0.92), suggesting that these pollutants share common sources and are likely controlled by similar atmospheric mechanisms. This strong connection is commonly linked to emissions from combustion, industrial sources, vehicular exhaust, and resuspension of road dust, all of which produce a wide spectrum of particle sizes. The observation is consistent with the previous research in South Asian industrial and urban areas, which reported a strong interrelation between PM_2.5_ and PM_10_ because of the similar sources of emissions and the low ability to disperse pollutants (Begum et al. 2011; Lu et al. 2021). Relative humidity (RH) was weakly positively correlated with PM_2.5_ (r = 0.20) and PM_10_ (r = 0.18); thus, it might not be a major factor driving PM concentrations, yet it can contribute to increases in particle concentrations under specific atmospheric conditions. Elevated RH may cause hygroscopic growth of particles and hence increase their mass and optical properties (Won et al. 2021). Also, high RH often accompanies stable layers in the atmosphere, which inhibit vertical mixing and keep contaminants near the surface. Such trends were supported by previous regional investigations, wherein high PM concentrations were linked to high humidity levels in winter months, caused both by larger particle size and by atmospheric stagnation (Tai et al. 2010; Zhang et al. 2013).

Temperature, on the other hand, showed a weak negative correlation (r = −0.19) with both PM_2.5_ and PM_10_, suggesting that lower temperatures may predispose to particulate pollution. More specifically, this negative correlation is mainly caused by temperature inversions, which often occur in colder months when a blanket of warm air over a region traps cooler air, preventing pollutants near the surface from mixing. Moreover, lower temperatures are usually accompanied by greater use of heating fuels, such as biomass and coal, which adds to particulate matter emissions. The results agree with the worldwide and regional literature reporting the increased PM concentrations in winter and autumn in cities and industrial areas (Salam et al. 2003; Seinfeld and Pandis 2016).

### Wind characteristics and influence on particulate matter dispersion

Meteorological factors substantially influence the spatial distribution of particulate matter, as relative humidity and temperature may exhibit weak correlations with PM concentration, whereas wind velocity and direction are more critical factors of dispersion. The prevalent wind in the industrial and residential areas was oriented along the east–west axis, with speeds ranging from 2.2 to 2.9 m/s and 2.1 to 2.9 m/s, respectively. Despite these wind speeds being categorised as moderate and typically favourable for pollutant dispersion, geographical analysis consistently indicated that the highest concentrations of PM_2.5_ and PM_10_ were located in the far west of both regions. The trend indicates that pollutants generated in the east were transported by downstream air currents and then deposited or accumulated in the western regions. This mode of directional transmission is aligned with the findings of (Molina et al. 2010) as the study has addressed the significance of wind corridors in determining pollution paths. The data presented herein correlate with the synoptic circulation over Bangladesh, which during the dry season is dominated by north-westerly and westerly winds (Climate—Banglapedia 2021).

However, multiple deviations were observed in specific locations such as Battail, Khajanagar, and Ailchara Bazar, where the wind flow deviated from the prevailing direction. The disparities can be attributed to local environmental conditions, including vegetation cover, built structure, and terrain changes (Zhang et al. 2013). Additional phenomena, such as urban heat and thermally induced circulations in densely built environments, can further distort wind patterns, resulting in reverse or circular flows that disperse pollutants against the prevailing wind direction (Arnfield 2003). The results of the dispersion analysis illustrate significant differences in the dispersion of PM_2.5_ and PM_10_, with the latter travelling farther than the former. This finding is consistent with previously conducted studies inferring that PM_10_ is more mechanically dispersible, especially those relating to the movement of vehicles (Vehicular), given that it has a greater aerodynamic size (Gulia et al. 2015). PM_10_ after resuspension and transportation is likely to carry more momentum than other particles and can even travel long distances in areas with high traffic or industrial activity, particularly under preferred meteorological conditions (Querol et al. 2004). Conversely, PM_2.5,_ as the smaller, lighter particles, are suspended longer, but their exposure to atmospheric instability, moisture, and chemistry is more critical. This vulnerability to weather conditions may limit its horizontal dispersion, even with long residence times in the atmosphere (WHO 2017). The results reveal that while temperature and humidity exert secondary effects, wind parameters are the primary determinants of PM transmission and regional variability. The combined effects of undisturbed atmospheric conditions, potential temperature inversion, and east–west wind patterns elucidate PM concentrations in downwind regions, underscoring the need to incorporate meteorological dynamics into air quality management systems in rapidly urbanising and industrialising areas.

### Gaussian dispersion modelling and distance travelling

The results from Fig. 6 demonstrate that particulate matter emissions from industrial and vehicle sources pose significant health hazards, both in the immediate industrial vicinity and in adjacent residential areas. The dispersion research indicates that particulate matter disperses over significant distances, extending beyond industrial areas. As a result, residential populations, although geographically distanced from direct emission sources, are already facing heightened exposure levels. This indicates that health problems are not limited to workers or nearby populations but are spreading to wider areas, raising significant concerns about long-term public health effects. The model-based analysis simultaneously demonstrates significant spatial variations in dispersion distances among sampling sites, indicating considerable local heterogeneity in emission intensity and environmental factors. Both PM_2.5_ and PM_10_ demonstrated peak dispersion at sampling site 32, signifying a possible pollution hotspot. This is probably linked to concentrated rice mills, traffic congestion, and heat-producing industries, which have been shown to emit intricate plumes containing both coarse and fine particles that disperse extensively due to turbulence and wind dynamics (Begum and Hopke 2018; Brook et al. 2004). Conversely, sample locations 7 and 33 exhibited significantly reduced dispersion distances. The restricted dispersion may be ascribed to diminished emission intensity, wind obstructions caused by flora or constructed entities, and localised microclimatic conditions that hinder particulate transport (Wang et al. 2024). These results highlight that PM dispersion poses significant health hazards in residential settings, with dispersion levels heavily influenced by local emission sources and environmental factors. PM₁₀ exhibits greater apparent dispersion than PM₂.₅ across the study area. This pattern is not attributed to intrinsic particle-size transport properties, but rather to site-specific and source-dominated processes governing near-field particulate dynamics. In particular, the enhanced spatial spread of PM₁₀ is likely driven by (i) resuspension of coarse particles from unpaved or dust-rich surfaces induced by vehicular movement (Linda et al. 2025), (ii) stronger near-ground turbulence generated by traffic flow and industrial activities within the study area (Pospisil et al. 2020), and (iii) spatial heterogeneity in emission sources, including localized coarse particulate emissions from rice mills and road dust (Fan et al. 2021). Collectively, these mechanisms can intermittently increase the mobility and redistribution of PM₁₀ within the local environment, leading to elevated short-range dispersion, even though its long-range atmospheric transport potential is generally lower than that of PM₂.₅. In contrast, PM₂.₅, due to its finer aerodynamic diameter, remains suspended for longer periods and is more efficiently transported over longer distances with relatively smoother spatial gradients, resulting in more uniform dispersion patterns but lower localized variability. Furthermore, the observed contrast between concentration peaks and troughs across sampling locations **(**Fig. 6a**)** indicates strong spatial variability in emission intensity and micro-environmental conditions, which further modulate the near-field transport behavior of both PM fractions. While Fig. 6b highlights that mixed emissions from the sources disperse into nearby residential areas, where PM_2.5_ and PM_10_ accumulate. Since PMs travel farther, residents located even farther from pollution sources remain vulnerable to exposure, thereby amplifying potential health risks. The results support the relevance of considering the micro-environmental conditions (land cover, surface roughness, and topographic limitations) in modelling urban and peri-urban PM_10_ air pollution.

### Air quality and non-carcinogenic health risks concern

Our findings indicate that PM_2.5_ can travel considerable distances from the emission source, exposing populations not only near rice processing hubs but also in surrounding residential areas. The behaviour of PM_2.5_ and PM_10_ dispersion is crucial for public health. PM_2.5_ particles are particularly dangerous because they can penetrate deeply into the respiratory system, reaching the alveoli and entering the bloodstream, potentially causing cardiovascular diseases, irreversible respiratory conditions, and premature mortality (WHO 2021a, b). PM_10_, while primarily deposited in the upper respiratory tract, can still cause respiratory irritation and exacerbate asthma, bronchitis, and allergic diseases, particularly among vulnerable groups such as children, the elderly, and individuals with pre-existing respiratory conditions (Lelieveld et al. 2015; WHO 2018).

In this study, all 35 industrial-zone sampling points exceeded the PM_2.5_ Hazard Quotient (HQ) threshold (max. 3.98), indicating substantial non-carcinogenic risk, and 33 residential sites also surpassed the safe limit (max. 4.72), suggesting that prolonged exposure affects populations beyond industrial areas. These results are consistent with findings from other regional studies (Saju et al. 2023; Nair et al. 2011) and highlight that sources of PM pollution are not limited to industrial activity but include vehicle emissions, biomass burning, construction dust, and open waste burning (Onyango et al. 2024; Simões Amaral et al. 2016).

Moreover, the observed non-carcinogenic risks align with global evidence on the effects of PM_2.5_ exposure, including asthma, chronic obstructive pulmonary disease (COPD), heart disease, stroke, and atherosclerosis (Alexeeff et al. 2021; Krittanawong et al. 2023; Sinkemani et al. 2018). Importantly, the outcomes of this study go beyond a purely local perspective by integrating airborne PM hotspot mapping and health risk assessment, providing insight into how PM emissions from rice processing mills can affect surrounding communities. These results contribute to the broader scientific understanding of PM exposure in climate-vulnerable regions worldwide, informing both local mitigation strategies and global discussions on particulate pollution management.

### Air Q + tools-based health risks

Knowledge of the health risks associated with air pollutants is crucial for understanding their impacts on public health and the environment. Using AirQ +, the study estimated health risks of air pollutants in Kushtia’s industrial hub, including lung cancer, COPD, IHD, stroke (30 +), and respiratory infections (ages 0–5).

#### Acute lower respiratory infection (ALRI) in children

The overall estimated percentage of mortality attributed to prolonged exposure to PM_2.5_ among children (< 5 years) due to ALRI in residential areas is up to 48.99. The estimated number of attributable cases per 100,000 at-risk population is max. of 0.81. The ALRI associated with airborne particulate matter is a significant health concern, particularly in densely populated and industrialised regions. The global Burden of Disease (GBD) 2019 study, conducted by its risk factor collaborators, highlighted PM_2.5_ as a major contributor to respiratory mortality on a global scale (Collaborators and Ärnlöv, 2020). Children are more susceptible to the harmful effects of air pollution on their developing lungs, which remain highly vulnerable during early life. Factors such as lower oxidative detoxification efficiency, higher metabolic and breathing rates, and increased oxygen consumption heighten their risks (Dondi et al. 2023). Because these fine particulate matters can easily infiltrate and penetrate deep into children’s lungs, leading to inflammation and a reduced ability of the lungs to combat infections (Zhang et al. 2023). Some research findings indicate that annual mortality rates have notable health consequences for young children, mostly due to ALRI. A study by Maji et al. (2018) found that PM_2.5_ exposure in Dhaka was estimated to cause 9,051 premature deaths. Among these, 201 fatalities (95% Cl: 137–248) were attributed to ALRI. Notably, a higher prevalence of respiratory symptoms was observed among rice mill workers who logged over 10 h of daily labour and those with more than 15 years of occupational exposure, with incidence rates of 41.3% and 39%, respectively (Choudhury et al. 2023). According to Siddique et al. (2024), 49.6% of rice mill workers had respiratory symptoms, and 39.1% had a history of respiratory infections.

#### Chronic obstructive pulmonary disease (COPD) for adults

The findings of this study highlight the significant burden of COPD-related mortality attributable to prolonged exposure to PM_2.5_ among adults. When examining the absolute number of deaths attributed to COPD due to PM_2.5_ exposure, residential areas reported 2 deaths, while industrial areas experienced a single death only. Although the death case is lower, the results underscore the pervasive impact of air pollution on respiratory health across different environments. In residential areas, the estimated proportion of deaths linked to PM_2.5_ exposure was 48.01%, slightly higher than the corresponding proportion in industrial areas (43.47%). Additionally, the estimated number of attributable cases per 100,000 at-risk population was higher in residential areas (3.02) than in industrial areas (2.73). In urban Bangladesh, the impact of air pollution on public health is now a concerning issue. The cohort study provides compelling evidence of a link between prolonged exposure to elevated air pollutant levels and a heightened risk of developing COPD, and the short-term exposure to air pollution increased hospital admissions (Khan et al. 2019). A study conducted by Maji et al. (2018) found that PM_2.5_ exposure in Dhaka led to an estimated 9,051 premature deaths. Among these, 1246 were attributed to COPD. According to Siddharthan et al. (2018), exposure to household air pollution has been linked to increased gen-specific prevalence of COPD, especially among women, and is likely one of the primary population-attributable risk factors for COPD in low-resource environments, with estimated population-attributable risks of 1.70 in women and 1.21 in men.

#### Ischemic heart disease (IHD) for adults

The results illustrate the estimated mortality rates from ischemic heart disease (IHD) among individuals aged 30 + years due to prolonged exposure to PM_2.5_ and PM_10_. Residential areas show slightly higher mortality rates and risks than industrial areas for both PM_2.5_ and PM_10_. In residential areas, the mortality rate due to IHD from prolonged exposure to PM_2.5_ is estimated to range from 45.59% to 64.6%, corresponding to approximately 7 deaths; in industrial areas, the range is 45.55% to 64.53%, leading to around 2 deaths. By contrast, a similar estimated mortality rate for IHD was observed in both industrial and residential areas. Misra et al. (2007) found that prolonged exposure to PM has a more pronounced effect on cardiovascular mortality than short-term exposure. The study revealed that increased PM was associated with a 23% higher risk of IHD mortality and a 24% higher risk of cerebrovascular mortality (Raihan et al. 2024), values that are lower than those reported in the current study. According to the latest WHO data in 2021, coronary heart disease deaths in Bangladesh reached 118,287, or 15.23% of total deaths, which are mostly influenced by air pollution (Saquib et al. 2012).

#### Stroke for adults

The findings of this research indicate a substantial health burden associated with prolonged exposure to PM_2.5_ and stroke-related mortality among adults aged 30 + years. Despite 72.45% observed in residential areas and 72.15% in industrial areas, the absolute number of deaths differed markedly between the two. Residential areas experienced around 7 deaths, while industrial areas had 2 deaths. Furthermore, the estimated number of attributable cases, 10.23 in residential areas and 10.20 in industrial areas, provides a more standardised perspective of the impact of PM_2.5_ exposure. A study investigated that around 34% of strokes, 26% of ischemic heart disease, 22% of chronic obstructive pulmonary disease, and 6% of lung cancer were attributed to indoor air pollution (Landrigan 2017). However, globally, approximately 1,989,686 stroke deaths (95% UI: 1,530,479–2,493,238) were attributable to air pollution in 2021 (Fan et al. 2024). Exposure to PM_2.5_ triggers chronic inflammation and arterial blockages in adults. This damage to the inner lining of blood vessels (endothelial dysfunction), the formation of fatty deposits in arteries (atherosclerosis), increased platelet activity, and a higher tendency for blood to clot are critical risk factors for stroke (Lee et al. 2018).

#### Lung cancer (LC) for adults

The key findings indicate a significant difference in mortality risk associated with prolonged exposure to PM_2.5_ and PM_10_ among individuals aged 30 + years due to lung cancer. While PM_2.5_ exposure results in mortality percentages of 34.16% in residential and 33.03% in industrial areas, no actual deaths were recorded. In contrast, PM_10_ exposure presents a much higher estimated mortality percentage, 65.79% in residential and 58.2% in industrial areas, leading to 1 death only in residential areas, which explains that lung cancer in adults does not seem to be triggered by PM exposure solely, although these are lung carcinogens; other factors influence it. Wang et al. (2025) noted that PM_10_ sometimes contains more carcinogenic and inflammatory components (crustal metals, silica, etc.) than PM_2.5_. Again, mortality rates per 100,000 individuals reinforce this trend, showing that PM_10_ exposure contributes more significantly to lung cancer risk (0.86 in residential vs. 0.76 in industrial areas) compared to PM_2.5_ exposure (0.44 and 0.43, respectively).

## Limitations and prospective research direction

This study has acknowledged a few shortcomings. There was a lack of capacity for effective seasonal monitoring and measurement of PM concentration variability, which may have led to an incomplete understanding or a less robust scenario of the phenomena under investigation. Besides, the relatively short winter-based sampling period may not fully capture seasonal variability in PM transport from agricultural regions. Long-term monitoring across multiple seasons, particularly periods when predominant winds originate from agricultural areas, would provide a more robust assessment of the relationships among PM concentrations, meteorological parameters (humidity and temperature), and source regions. Seasonal phenomena such as winter inversions, monsoon rainfall, and post-monsoon humidity are expected to substantially influence PM dispersion and deposition patterns, and future studies incorporating year-round sampling and wind-direction–focused analysis are therefore recommended.

Measurement uncertainty and bias are general limitations of OPC-based measurements, particularly under high-humidity conditions, because hygroscopic particle growth and associated optical scattering effects cannot be ignored. However, our measurements were conducted exclusively during the winter season (December), when ambient relative humidity was comparatively low and atmospheric conditions were dry and stable, indicating minimal humidity-driven influence on the observed particulate matter levels. Additionally, the study relied solely on device-generated data without any cross-validation using satellite imagery, which may have had a minor effect on the overall reliability of the results. To enhance the robustness of future research, it is recommended to validate device-based outcomes by comparing them with satellite data or other independent sources.

In addition, a limitation of this study is the inability to isolate the specific contribution of rice processing mills to ambient PM pollution, as other sources, including vehicular traffic, industrial activities, agricultural residue burning, and biomass combustion, may also have influenced the observed concentrations. Consequently, the findings represent the cumulative effects of mixed-source air pollution rather than rice mill emissions alone. Furthermore, the absence of a comparable rural control site without rice milling activities limited the establishment of a true background baseline and the clear differentiation of rice mill-derived emissions from other local sources. Future studies should incorporate advanced source apportionment techniques, such as Positive Matrix Factorization (PMF), along with control and comparative sampling sites, to better quantify source-specific contributions and associated health risks in rice mill-dominated regions.

Further, although short-term intensive monitoring during critical high-exposure periods is a widely accepted approach in environmental health and air quality research to identify peak-risk conditions and support targeted mitigation strategies, the inclusion of multi-seasonal data would undoubtedly strengthen the understanding of temporal variability, extended monitoring campaigns were beyond the logistical and financial scope of the present investigation. Thus, we have highlighted the necessity of future multi-seasonal investigations, including monsoon and summer observations, to better quantify seasonal dispersion dynamics and provide a more comprehensive annual health risk assessment.

Furthermore, a limitation of this study is the absence of a comprehensive historical dataset, which restricted long-term trend analysis and limited the development of robust predictive models for improved forecasting and decision-making. In addition, the study lacked direct field-based health outcome data, including cause-specific mortality, hospital admissions, and hospital visits. The unavailability of disease-specific hospital records and community health survey data also prevented direct validation of AirQ + -estimated health impacts against observed clinical evidence. Addressing these data gaps in future research would enable a more comprehensive assessment of particulate matter exposure and its health consequences.

## Conclusion

This study provides a comprehensive, data-driven evaluation of PM emissions from rice mills in the Kushtia District, Bangladesh, highlighting their significant impact on ambient air quality and public health. The assessment integrates spatial distribution analysis, meteorological influences, dispersion dynamics, and quantitative health risk estimation for residents. The assessment reveals an alarming prevalence of PM_2.5_ and PM_10_ pollutants across industrial and residential areas, exceeding national, WHO, and EPA air quality standards. Interestingly, dispersion modelling indicates that PM emissions are not spatially confined; westerly winds transport pollutants from eastern sources to downwind residential areas, increasing exposure risks. The Hazard Quotient indicated very high non-carcinogenic health risks from PM_2.5_ exposure at all sampled locations. In contrast, WHO endorsed the AirQ + tool, which elevated disease-specific mortality and morbidity rates for ALRI in children and COPD, IHD, and Stroke in adults. PM_10_ exposure was notably associated with increased Lung Cancer mortality in adults. These findings demonstrate that PM from rice mills adversely affects both workers and surrounding communities. However, further research is needed to quantify cause-specific mortality, hospitalisations, and clinic visits to better characterise the health burden. Winter meteorological conditions in Bangladesh during December may enhance pollutant accumulation and dispersion; therefore, the measured concentrations and associated health risks likely represent a worst-case winter scenario rather than annual conditions. Future studies should include multi-seasonal monitoring to better capture seasonal variations in pollutant dispersion and health risks. The results highlight the need for technically grounded, field-relevant mitigation measures tailored to rice-husk–based boiler systems and rice milling activities, as well as stronger occupational health protection. Engineering controls such as cyclone separators for coarse particulate removal, baghouse (fabric) filters for fine particulate capture, and improved combustion system designs to enhance fuel efficiency and reduce incomplete combustion emissions. In addition, we emphasize operational and process-level interventions, including improved enclosure of material-handling and dust-generating units, to minimize fugitive emissions within the mill premises. Continuous monitoring and development of mitigation technologies are essential to protect public health and ensure a sustainable future for residents.

## Supplementary Information

Below is the link to the electronic supplementary material.

Supplementary file1 (DOCX 125 KB)

## Data Availability

All data generated or analysed in this study are presented within this manuscript in the form of tables, figures, and supplementary materials. Additional raw data or supporting information are available from the authors upon reasonable request.
