# Supplementary material for: Integrated spatial analysis and Gaussian dispersion modelling of airborne particulate matter from rice processing mill hubs in rural Bangladesh and associated public health risks
Source: Environ Sci Pollut Res Int. 2026 Jul 22;33(24):12319–41. doi: 10.1007/s11356-026-38036-9 (PMC13468869; doi:10.1007/s11356-026-38036-9)
Supplement: Supplementary file 1 [file 11356_2026_38036_MOESM1_ESM.docx]

**Integrated Spatial Analysis and Gaussian Dispersion Modelling of Airborne Particulate Matter from Rice Processing Mill Hubs in Rural Bangladesh and Associated Public Health Risks**

Rubaiatul Islam Zerin ^a^, Md. Kamrul Hossain ^a^, Humaira Rashid ^a^, Sababa Tasnim ^b^, Md. Julfikar Ali ^c^, Md. Mizanur Rahman^d^, Mohd. Maniruzzaman ^e^, Rafiquel Islam ^e, f*^

*^a^ Department of Environmental Science and Geography, Islamic University, Kushtia-7003, Bangladesh*

*^b^ Department of Environmental Science and Management, Independent University, Dhaka 1245, Bangladesh*

*^c^ Department of Information and Communication Technology, Islamic University, Kushtia 7003, Bangladesh*

*^d^ Department of Mathematics, Faculty of Science, Islamic University, Kushtia 7003, Bangladesh*

*^e^ Department of Applied Chemistry and Chemical Engineering, Islamic University, Kushtia 7003, Bangladesh*

*^f^ School of Environmental and Life Sciences, The University of Newcastle, Callaghan, NSW 2308, Australia*

****Corresponding author:*** Dr. Rafiquel Islam; Email: [rafiquel.islam@newcastle.edu.au](mailto:rafiquel.islam@newcastle.edu.au); [rafiquel.islam@kfupm.edu.sa](mailto:rafiquel.islam@kfupm.edu.sa)

ORCID:  [0000-0002-3033-4840](javascript:popup_orcidDetail('https://orcid.org'%20,'0000-0002-3033-4840');)

**Table S1(a):** Measured particulate matter concentration in the Industrial area (1st and 2^nd^ week)

| **FID** | **PM _2.5_ (µg/m^3^)** | | | | | | **PM _10_ (µg/m^3^)** | | | | | | **RH(%)** | **Temp.(^0^C)** |
| --- | --- | --- | --- | --- | --- | --- | --- | --- | --- | --- | --- | --- | --- | --- |
|  | **Morning** | | | **Evening** | | | **Morning** | | | **Evening** | | |  |  |
|  | **HT 9600** | **HS 5003** | **Avg.** | **HT 9600** | **HS 5003** | **Avg.** | **HT 9600** | **HS 5003** | **Avg.** | **HT 9600** | **HS 5003** | **Avg.** |  |  |
| 0 | 96 | 101 | **98.5** | 81 | 88 | **84.5** | 122 | 127 | **124.5** | 105 | 112 | **108.5** | 56.7 | 26.23 |
| 1 | 77 | 83 | **80** | 81 | 89 | **85** | 98 | 103 | **100.5** | 101 | 105 | **103** | 52.2 | 27.88 |
| 2 | 67 | 62 | **64.5** | 72 | 67 | **69.5** | 83 | 76 | **79.5** | 87 | 83 | **85** | 50.2 | 28.3 |
| 3 | 89 | 83 | **86** | 94 | 88 | **91** | 134 | 127 | **130.5** | 138 | 132 | **135** | 47 | 30.42 |
| 4 | 59 | 66 | **62.5** | 41 | 48 | **44.5** | 75 | 83 | **79** | 52 | 56 | **54** | 43.5 | 30.82 |
| 5 | 53 | 61 | **57** | 42 | 50 | **46** | 68 | 74 | **71** | 58 | 63 | **60.5** | 45.6 | 30.89 |
| 6 | 54 | 62 | **58** | 52 | 59 | **55.5** | 67 | 73 | **70** | 66 | 72 | **69** | 43 | 32.37 |
| 7 | 60 | 56 | **58** | 69 | 64 | **66.5** | 74 | 73 | **73.5** | 80 | 75 | **77.5** | 43.4 | 31.97 |
| 8 | 68 | 72 | **70** | 105 | 111 | **108** | 94 | 101 | **97.5** | 135 | 142 | **138.5** | 45.5 | 31.14 |
| 9 | 52 | 56 | **54** | 48 | 53 | **50.5** | 65 | 72 | **68.5** | 68 | 74 | **71** | 47.8 | 29.8 |
| 10 | 62 | 68 | **65** | 40 | 47 | **43.5** | 84 | 91 | **87.5** | 55 | 59 | **57** | 45.1 | 30.65 |
| 11 | 59 | 63 | **61** | 61 | 65 | **63** | 77 | 82 | **79.5** | 84 | 92 | **88** | 41.5 | 34.6 |
| 12 | 48 | 53 | **50.5** | 48 | 53 | **50.5** | 62 | 67 | **64.5** | 67 | 63 | **65** | 39.5 | 33.19 |
| 13 | 64 | 72 | **68** | 40 | 47 | **43.5** | 99 | 103 | **101** | 50 | 56 | **53** | 38.6 | 33.5 |
| 14 | 46 | 53 | **49.5** | 48 | 53 | **50.5** | 57 | 62 | **59.5** | 62 | 67 | **64.5** | 39.9 | 33.7 |
| 15 | 49 | 55 | **52** | 39 | 46 | **42.5** | 63 | 68 | **65.5** | 55 | 59 | **57** | 39.1 | 34.45 |
| 16 | 47 | 53 | **51.5** | 52 | 58 | **55** | 59 | 62 | **60.5** | 70 | 75 | **72.5** | 44.6 | 31.31 |
| 17 | 48 | 42 | **45** | 47 | 53 | **50** | 62 | 57 | **59.5** | 63 | 68 | **65.5** | 41.6 | 31.73 |
| 18 | 61 | 55 | **58** | 46 | 53 | **49.5** | 79 | 72 | **75.5** | 64 | 79 | **71.5** | 42.4 | 32.6 |
| 19 | 72 | 78 | **75** | 79 | 84 | **81.5** | 95 | 92 | **93.5** | 98 | 104 | **101** | 40.6 | 32.7 |
| 20 | 84 | 87 | **85.5** | 90 | 87 | **88.5** | 106 | 113 | **109.5** | 123 | 119 | **121** | 42.3 | 33.2 |
| 21 | 91 | 85 | **88** | 86 | 89 | **87.5** | 113 | 108 | **110.5** | 109 | 113 | **111** | 40.7 | 34.04 |
| 22 | 78 | 84 | **81** | 72 | 65 | **68.5** | 93 | 98 | **95.5** | 89 | 84 | **86.5** | 37.5 | 34.29 |
| 23 | 83 | 90 | **86.5** | 73 | 79 | **76** | 107 | 114 | **110.5** | 99 | 103 | **101** | 38.3 | 33.9 |
| 24 | 72 | 67 | **69.5** | 76 | 83 | **79.5** | 96 | 92 | **94** | 110 | 117 | **113.5** | 40.2 | 32.3 |
| 25 | 48 | 54 | **51** | 41 | 47 | **44** | 62 | 69 | **65.5** | 54 | 59 | **56.5** | 42.9 | 31.3 |
| 26 | 86 | 81 | **83.5** | 106 | 112 | **109** | 117 | 112 | **114.5** | 131 | 127 | **129** | 42.6 | 32.34 |
| 27 | 52 | 57 | **54.5** | 45 | 41 | **43** | 68 | 75 | **71.5** | 58 | 63 | **60.5** | 38.6 | 34.15 |
| 28 | 45 | 52 | **48.5** | 47 | 53 | **50** | 60 | 65 | **62.5** | 63 | 60 | **61.5** | 39.9 | 32.33 |
| 29 | 40 | 46 | **43** | 61 | 67 | **64** | 51 | 58 | **54.5** | 79 | 72 | **75.5** | 40.1 | 32.5 |
| 30 | 44 | 41 | **42.5** | 40 | 47 | **43.5** | 56 | 51 | **53.5** | 54 | 49 | **51.5** | 38.6 | 32.2 |
| 31 | 110 | 115 | **112.5** | 250 | 257 | **253.5** | 154 | 163 | **158.5** | 358 | 364 | **361** | 37 | 33.46 |
| 32 | 42 | 49 | **45.5** | 44 | 53 | **48.5** | 67 | 73 | **70** | 54 | 61 | **57.5** | 43.6 | 30.09 |
| 33 | 41 | 47 | **44** | 47 | 43 | **45** | 57 | 62 | **59.5** | 61 | 57 | **59** | 43.1 | 30.9 |
| 34 | 44 | 48 | **46** | 39 | 45 | **42** | 65 | 71 | **68** | 57 | 62 | **59.5** | 43.9 | 29.7 |

**Table S1(b):** Measured particulate matter concentration in residential area (1st and 2^nd^ week)

| **FID** | **PM _2.5_ (µg/m^3^)** | | | | | | **PM _10_ (µg/m^3^)** | | | | | | **RH(%)** | **Temp.(^0^C)** |
| --- | --- | --- | --- | --- | --- | --- | --- | --- | --- | --- | --- | --- | --- | --- |
|  | **Morning** | | | **Evening** | | | **Morning** | | | **Evening** | | |  |  |
|  | **HT 9600** | **HS 5003** | **Avg.** | **HT 9600** | **HS 5003** | **Avg.** | **HT 9600** | **HS 5003** | **Avg.** | **HT 9600** | **HS 5003** | **Avg.** |  |  |
| 0 | 166 | 169 | **167.5** | 73 | 71 | **72** | 206 | 209 | **207.5** | 111 | 108 | **109.5** | 63 | 23.97 |
| 1 | 136 | 137 | **136.5** | 68 | 63 | **65.5** | 171 | 173 | **172** | 102 | 98 | **100** | 59.6 | 25.84 |
| 2 | 138 | 143 | **140.5** | 74 | 79 | **76.5** | 173 | 175 | **174** | 106 | 112 | **109** | 55.4 | 26.27 |
| 3 | 134 | 139 | **136.5** | 94 | 98 | **96** | 167 | 173 | **170** | 124 | 129 | **126.5** | 55.1 | 27.29 |
| 4 | 148 | 153 | **150.5** | 61 | 63 | **62** | 186 | 190 | **188** | 86 | 89 | **87.5** | 52.4 | 28.4 |
| 5 | 136 | 139 | **137.5** | 106 | 109 | **107.5** | 169 | 172 | **170.5** | 142 | 146 | **144** | 52.5 | 28.35 |
| 6 | 129 | 135 | **132** | 83 | 87 | **85** | 157 | 163 | **160** | 107 | 113 | **110** | 53.1 | 28.5 |
| 7 | 127 | 132 | **129.5** | 98 | 114 | **106** | 157 | 163 | **160** | 131 | 138 | **134.5** | 49.6 | 28.7 |
| 8 | 119 | 113 | **116** | 112 | 118 | **115** | 146 | 142 | **144** | 143 | 147 | **145** | 51.8 | 29.5 |
| 9 | 116 | 122 | **119** | 82 | 89 | **85.5** | 143 | 146 | **144.5** | 109 | 114 | **111.5** | 48.1 | 29.6 |
| 10 | 125 | 121 | **123** | 88 | 83 | **85.5** | 165 | 161 | **163** | 131 | 128 | **129.5** | 47.4 | 29.8 |
| 11 | 115 | 111 | **113** | 89 | 93 | **91** | 142 | 138 | **140** | 132 | 137 | **134.5** | 47.5 | 29.7 |
| 12 | 115 | 111 | **113** | 98 | 103 | **100.5** | 139 | 134 | **136.5** | 123 | 129 | **126** | 48.8 | 29 |
| 13 | 106 | 109 | **107.5** | 84 | 89 | **86.5** | 135 | 139 | **137** | 106 | 110 | **108** | 44.5 | 31.52 |
| 14 | 114 | 118 | **116** | 94 | 91 | **92.5** | 158 | 163 | **160.5** | 123 | 119 | **121** | 44 | 31.33 |
| 15 | 103 | 107 | **105** | 81 | 87 | **84** | 126 | 129 | **127.5** | 103 | 109 | **106** | 47.1 | 29.6 |
| 16 | 102 | 106 | **104** | 87 | 83 | **85** | 126 | 131 | **128.5** | 113 | 109 | **111** | 47 | 29.7 |
| 17 | 101 | 105 | **103** | 90 | 94 | **92** | 129 | 134 | **131.5** | 111 | 117 | **114** | 42.5 | 32.49 |
| 18 | 99 | 96 | **97.5** | 87 | 95 | **91** | 123 | 119 | **121** | 109 | 113 | **111** | 43.6 | 33.05 |
| 19 | 105 | 101 | **103** | 83 | 89 | **86** | 144 | 141 | **142.5** | 103 | 107 | **105** | 43.1 | 31.92 |
| 20 | 91 | 97 | **94** | 78 | 85 | **81.5** | 120 | 126 | **123** | 102 | 109 | **105.5** | 47.6 | 30.12 |
| 21 | 79 | 83 | **81** | 76 | 82 | **79** | 100 | 104 | **102** | 96 | 101 | **98.5** | 43.8 | 31.55 |
| 22 | 90 | 94 | **92** | 65 | 69 | **67** | 120 | 125 | **122.5** | 85 | 89 | **87** | 43.5 | 31.04 |
| 23 | 63 | 69 | **66** | 49 | 54 | **51.5** | 83 | 85 | **84** | 65 | 71 | **68** | 44.9 | 30.8 |
| 24 | 88 | 93 | **90.5** | 71 | 79 | **75** | 113 | 117 | **115** | 97 | 103 | **100** | 42.4 | 32.7 |
| 25 | 105 | 107 | **106** | 71 | 79 | **75** | 132 | 139 | **135.5** | 96 | 102 | **99** | 45 | 31.68 |
| 26 | 73 | 74 | **73.5** | 121 | 124 | **122.5** | 92 | 95 | **93.5** | 159 | 163 | **161** | 43.4 | 31.72 |
| 27 | 94 | 98 | **96** | 67 | 72 | **69.5** | 128 | 133 | **130.5** | 90 | 95 | **92.5** | 43.7 | 30.79 |
| 28 | 66 | 72 | **69** | 63 | 68 | **65.5** | 81 | 85 | **83** | 84 | 89 | **86.5** | 44.6 | 30.8 |
| 29 | 76 | 79 | **77.5** | 74 | 76 | **75** | 101 | 104 | **102.5** | 100 | 103 | **101.5** | 41.2 | 31.03 |
| 30 | 63 | 61 | **62** | 67 | 72 | **69.5** | 80 | 77 | **78.5** | 89 | 93 | **91** | 40.4 | 32.4 |
| 31 | 56 | 59 | **57.5** | 63 | 68 | **65.5** | 74 | 79 | **76.5** | 85 | 89 | **87** | 40.8 | 32.7 |
| 32 | 75 | 79 | **77** | 59 | 66 | **62.5** | 96 | 99 | **97.5** | 83 | 84 | **83.5** | 39.7 | 31.9 |

**Table S1(c):** Measured particulate matter concentration in Industrial area (3^rd^ and 4^th^ week)

| **FID** | **PM _2.5_ (µg/m^3^)** | | | | | | **PM _10_ (µg/m^3^)** | | | | | | **RH(%)** | **Temp.(^0^C)** |
| --- | --- | --- | --- | --- | --- | --- | --- | --- | --- | --- | --- | --- | --- | --- |
|  | **Morning** | | | **Evening** | | | **Morning** | | | **Evening** | | |  |  |
|  | **HT 9600** | **HS 5003** | **Avg.** | **HT 9600** | **HS 5003** | **Avg.** | **HT 9600** | **HS 5003** | **Avg.** | **HT 9600** | **HS 5003** | **Avg.** |  |  |
| 0 | 148 | 153 | **150.5** | 164 | 169 | **166.5** | 263 | 269 | **266** | 214 | 218 | **216** | 66.3 | 21.72 |
| 1 | 134 | 137 | **135.5** | 103 | 109 | **106** | 184 | 187 | **185.5** | 138 | 141 | **139.5** | 64.2 | 22.6 |
| 2 | 127 | 134 | **130.5** | 102 | 106 | **104** | 171 | 174 | **172.5** | 130 | 133 | **131.5** | 63.6 | 23.45 |
| 3 | 158 | 153 | **155.5** | 141 | 137 | **139** | 216 | 213 | **214.5** | 269 | n | **269** | 59.8 | 25.5 |
| 4 | 135 | 137 | **136** | 105 |  | **106** | 182 | 185 | **183.5** | 146 | 149 | **147.5** | 55.4 | 26.48 |
| 5 | 165 | 172 | **168.5** | 106 | 103 | **104.5** | 221 | 227 | **224** | 167 | 163 | **165** | 56.8 | 26.16 |
| 6 | 109 | 116 | **112.5** | 93 | 99 | **96** | 153 | 158 | **155.5** | 128 | 132 | **130** | 56.9 | 25.68 |
| 7 | 124 | 121 | **122.5** | 94 | 102 | **98** | 170 | 167 | **168.5** | 132 | 138 | **135** | 55.3 | 26.1 |
| 8 | 116 | 114 | **115** | 108 | 114 | **111** | 163 | 161 | **162** | 153 | 159 | **156** | 54.8 | 26.72 |
| 9 | 119 | 124 | **121.5** | 93 | 99 | **96** | 167 | 174 | **170.5** | 123 | 126 | **124.5** | 54.2 | 26.65 |
| 10 | 156 | 159 | **157.5** | 109 | 113 | **111** | 207 | 212 | **209.5** | 153 | 159 | **156** | 54.6 | 26.44 |
| 11 | 121 | 126 | **123.5** | 101 | 108 | **104.5** | 176 | 179 | **177.5** | 136 | 143 | **139.5** | 55.2 | 26.96 |
| 12 | 131 | 136 | **133.5** | 105 | 111 | **108** | 181 | 184 | **182.5** | 160 | 164 | **162** | 51.3 | 27.85 |
| 13 | 114 | 119 | **116.5** | 94 | 97 | **95.5** | 156 | 159 | **157.5** | 126 | 129 | **127.5** | 55.7 | 28.18 |
| 14 | 111 | 115 | **113** | 121 | 125 | **123** | 153 | 157 | **155** | 186 | 189 | **187.5** | 51.5 | 28.08 |
| 15 | 123 | 121 | **122** | 122 | 126 | **124** | 198 | 196 | **197** | 170 | 174 | **172** | 52.3 | 27.73 |
| 16 | 160 | 155 | **157.5** | 124 | 128 | **126** | 230 | 228 | **229** | 170 | 175 | **172.5** | 52.6 | 27.45 |
| 17 | 123 | 127 | **125** | 117 | 121 | **119** | 168 | 173 | **170.5** | 160 | 163 | **161.5** | 54.7 | 26.28 |
| 18 | 145 | 141 | **143** | 78 | 75 | **76.5** | 203 | 198 | **200.5** | 108 | 103 | **105.5** | 55.9 | 26.85 |
| 19 | 131 | 136 | **133.5** | 119 | 116 | **117.5** | 188 | 193 | **190.5** | 169 | 163 | **166** | 54.8 | 26.95 |
| 20 | 105 | 101 | **103** | 114 | 119 | **116.5** | 141 | 139 | **140** | 156 | 159 | **157.5** | 51.3 | 28.3 |
| 21 | 108 | 113 | **110.5** | 107 | 111 | **109** | 149 | 153 | **151** | 146 | 152 | **149** | 52.4 | 27.77 |
| 22 | 113 | 107 | **110** | 101 | 105 | **103** | 157 | 153 | **155** | 145 | 151 | **148** | 50.4 | 28.74 |
| 23 | 116 | 119 | **117.5** | 119 | 123 | **121** | 164 | 169 | **166.5** | 167 | 173 | **170** | 49.6 | 29.25 |
| 24 | 130 | 136 | **133** | 110 | 115 | **112.5** | 175 | 179 | **177** | 155 | 159 | **157** | 51 | 28.35 |
| 25 | 109 | 113 | **111** | 104 | 101 | **102.5** | 147 | 151 | **149** | 141 | 138 | **139.5** | 50.1 | 29.18 |
| 26 | 123 | 127 | **125** | 124 | 126 | **125** | 178 | 182 | **180** | 172 | 177 | **174.5** | 49.6 | 28.79 |
| 27 | 128 | 131 | **129.5** | 111 | 107 | **109** | 186 | 189 | **187.5** | 152 | 148 | **150** | 52.7 | 27.3 |
| 28 | 144 | 147 | **145.5** | 102 | 106 | **104** | 206 | 212 | **209** | 145 | 149 | **147** | 55.6 | 26.44 |
| 29 | 108 | 104 | **106** | 81 | 85 | **83** | 149 | 147 | **148** | 115 | 118 | **116.5** | 54.8 | 26.86 |
| 30 | 138 | 132 | **135** | 152 | 157 | **154.5** | 197 | 192 | **194.5** | 215 | 217 | **216** | 53.9 | 27.3 |
| 31 | 96 | 101 | **98.5** | 91 | 95 | **93** | 132 | 138 | **135** | 121 | 126 | **123.5** | 54.2 | 29.6 |
| 32 | 102 | 107 | **104.5** | 87 | 89 | **88** | 164 | 169 | **166.5** | 117 | 179 | **148** | 53.1 | 29.3 |
| 33 | 178 | 184 | **181** | 123 | 126 | **124.5** | 243 | 242 | **242.5** | 179 | 182 | **180.5** | 54.7 | 27.07 |
| 34 | 147 | 142 | **144.5** | 136 | 131 | **133.5** | 211 | 208 | **209.5** | 207 | 201 | **204** | 56.9 | 28.56 |

**Table S1(d):** Measured particulate matter concentration in the residential area (3^rd^ and 4^th^ week)

| **FID** | **PM _2.5_ (µg/m^3^)** | | | | | | **PM _10_ (µg/m^3^)** | | | | | | **RH(%)** | **Temp.(^0^C)** |
| --- | --- | --- | --- | --- | --- | --- | --- | --- | --- | --- | --- | --- | --- | --- |
|  | **Morning** | | | **Evening** | | | **Morning** | | | **Evening** | | |  |  |
|  | **HT 9600** | **HS 5003** | **Avg.** | **HT 9600** | **HS 5003** | **Avg.** | **HT 9600** | **HS 5003** | **Avg.** | **HT 9600** | **HS 5003** | **Avg.** |  |  |
| 0 | 160 | 154 | **157** | 102 | 96 | **99** | 219 | 217 | **218** | 156 | 152 | **154** | 63.1 | 20.12 |
| 1 | 146 | 153 | **149.5** | 98 | 94 | **96** | 198 | 189 | **193.5** | 111 | 131 | **121** | 65 | 20.65 |
| 2 | 143 | 138 | **140.5** | 112 | 115 | **113.5** | 192 | 194 | **193** | 154 | 143 | **148.5** | 59.2 | 22.2 |
| 3 | 137 | 142 | **139.5** | 97 | 96 | **96.5** | 187 | 180 | **183.5** | 109 | 95 | **102** | 56 | 22.77 |
| 4 | 143 | 137 | **140** | 123 | 128 | **125.5** | 194 | 204 | **199** | 183 | 198 | **190.5** | 56.3 | 23.03 |
| 5 | 131 | 140 | **135.5** | 129 | 136 | **132.5** | 180 | 187 | **183.5** | 189 | 197 | **193** | 57.1 | 22.55 |
| 6 | 146 | 144 | **145** | 121 | 121 | **121** | 200 | 207 | **203.5** | 193 | 199 | **196** | 59.4 | 21.57 |
| 7 | 140 | 132 | **136** | 145 | 143 | **144** | 190 | 198 | **194** | 201 | 194 | **197.5** | 58.5 | 22.42 |
| 8 | 139 | 137 | **138** | 134 | 125 | **129.5** | 187 | 177 | **182** | 167 | 181 | **174** | 58.7 | 22.44 |
| 9 | 145 | 149 | **147** | 128 | 136 | **132** | 193 | 200 | **196.5** | 172 | 186 | **179** | 55.6 | 22.17 |
| 10 | 141 | 146 | **143.5** | 123 | 113 | **118** | 194 | 187 | **190.5** | 166 | 152 | **159** | 52.3 | 23.33 |
| 11 | 158 | 157 | **157.5** | 102 | 109 | **105.5** | 206 | 212 | **209** | 174 | 162 | **168** | 51.8 | 25.03 |
| 12 | 151 | 151 | **151** | 143 | 140 | **141.5** | 208 | 205 | **206.5** | 222 | 242 | **232** | 49.4 | 24.8 |
| 13 | 148 | 145 | **146.5** | 130 | 129 | **129.5** | 200 | 207 | **203.5** | 198 | 198 | **198** | 47 | 26.58 |
| 14 | 152 | 157 | **154.5** | 137 | 147 | **142** | 207 | 211 | **209** | 176 | 169 | **172.5** | 48.5 | 26.7 |
| 15 | 134 | 124 | **129** | 132 | 136 | **134** | 180 | 190 | **185** | 165 | 165 | **165** | 51.2 | 26.63 |
| 16 | 103 | 97 | **100** | 111 | 115 | **113** | 200 | 201 | **200.5** | 149 | 152 | **150.5** | 50 | 25.99 |
| 17 | 138 | 131 | **134.5** | 156 | 154 | **155** | 186 | 193 | **189.5** | 202 | 198 | **200** | 50.2 | 26.45 |
| 18 | 137 | 144 | **140.5** | 107 | 108 | **107.5** | 183 | 185 | **184** | 148 | 141 | **144.5** | 51.7 | 25.92 |
| 19 | 125 | 131 | **128** | 122 | 127 | **124.5** | 189 | 194 | **191.5** | 164 | 174 | **169** | 50.9 | 24.86 |
| 20 | 134 | 144 | **139** | 126 | 118 | **122** | 179 | 170 | **174.5** | 176 | 195 | **185.5** | 51.8 | 25.38 |
| 21 | 151 | 157 | **154** | 131 | 127 | **129** | 201 | 198 | **199.5** | 191 | 201 | **196** | 49.8 | 26.72 |
| 22 | 128 | 132 | **130** | 112 | 102 | **107** | 167 | 159 | **163** | 154 | 163 | **158.5** | 51.4 | 27.54 |
| 23 | 161 | 165 | **163** | 143 | 134 | **138.5** | 227 | 226 | **226.5** | 195 | 187 | **191** | 47.4 | 25.94 |
| 24 | 106 | 103 | **104.5** | 87 | 84 | **85.5** | 167 | 158 | **162.5** | 127 | 129 | **128** | 44.1 | 27.24 |
| 25 | 207 | 213 | **210** | 165 | 174 | **169.5** | 263 | 268 | **265.5** | 201 | 220 | **210.5** | 42.5 | 29.92 |
| 26 | 286 | 296 | **291** | 178 | 171 | **174.5** | 368 | 378 | **373** | 267 | 265 | **266** | 46.7 | 30.15 |
| 27 | 122 | 114 | **118** | 110 | 101 | **105.5** | 159 | 153 | **156** | 161 | 181 | **171** | 47.9 | 27.09 |
| 28 | 150 | 157 | **153.5** | 123 | 117 | **120** | 213 | 214 | **213.5** | 174 | 158 | **166** | 53.1 | 26.9 |
| 29 | 111 | 108 | **109.5** | 107 | 104 | **105.5** | 161 | 155 | **158** | 143 | 139 | **141** | 51.2 | 27.68 |
| 30 | 161 | 157 | **159** | 156 | 158 | **157** | 226 | 223 | **224.5** | 201 | 218 | **209.5** | 47.2 | 29.96 |
| 31 | 145 | 143 | **144** | 134 | 128 | **131** | 202 | 209 | **205.5** | 187 | 201 | **194** | 54.2 | 27.57 |
| 32 | 96 | 97 | **96.5** | 101 | 95 | **98** | 132 | 129 | **130.5** | 151 | 148 | **149.5** | 53.7 | 28.73 |
| PM: Particulate Matter; Unit: µg/m^3^; Avg: Average; RH: Relative Humidity (%); Temp. Temperature (^0^C) | | | | | | | | | | | | | | |

**Table S2(a):** Average values of measured Particulate Matter Concentrations in the industrial area

| **FID** | **Latitude** | **Longitude** | **PM _2.5_ (µg/m^3^)** | **PM_10_(µg/m^3^)** | **Relative Humidity (%)** | **Temperature (^0^C)** | **Wind Speed (m/s)** |
| --- | --- | --- | --- | --- | --- | --- | --- |
| 0 | 23.88 | 89.09 | 125.00 | 178.75 | 61.5 | 23.975 | 2.75593 |
| 1 | 23.88 | 89.09 | 101.63 | 132.13 | 58.2 | 25.24 | 2.78477 |
| 2 | 23.88 | 89.08 | 92.13 | 117.13 | 56.9 | 25.875 | 2.85448 |
| 3 | 23.88 | 89.09 | 117.88 | 187.25 | 53.4 | 27.96 | 2.7895 |
| 4 | 23.88 | 89.09 | 87.25 | 116.00 | 49.45 | 28.65 | 2.80626 |
| 5 | 23.88 | 89.09 | 94.00 | 130.13 | 51.2 | 28.525 | 2.84037 |
| 6 | 23.88 | 89.08 | 80.50 | 106.13 | 49.95 | 29.025 | 2.84842 |
| 7 | 23.87 | 89.08 | 86.25 | 113.63 | 49.35 | 29.035 | 2.84383 |
| 8 | 23.87 | 89.08 | 101.00 | 138.50 | 50.15 | 28.93 | 2.81499 |
| 9 | 23.87 | 89.08 | 80.50 | 108.63 | 51 | 28.225 | 2.78416 |
| 10 | 23.87 | 89.07 | 94.25 | 127.50 | 49.85 | 28.545 | 2.67293 |
| 11 | 23.87 | 89.07 | 88.00 | 121.13 | 48.35 | 30.78 | 2.56296 |
| 12 | 23.87 | 89.07 | 85.63 | 118.50 | 45.4 | 30.52 | 2.47249 |
| 13 | 23.87 | 89.07 | 80.88 | 109.75 | 47.15 | 30.84 | 2.37824 |
| 14 | 23.87 | 89.07 | 84.00 | 116.63 | 45.7 | 30.89 | 2.29943 |
| 15 | 23.87 | 89.07 | 85.13 | 122.88 | 45.7 | 31.09 | 2.47789 |
| 16 | 23.87 | 89.06 | 97.50 | 133.63 | 48.6 | 29.38 | 2.44363 |
| 17 | 23.87 | 89.06 | 84.75 | 114.25 | 48.15 | 29.005 | 2.34618 |
| 18 | 23.87 | 89.06 | 81.75 | 113.25 | 49.15 | 29.725 | 2.25611 |
| 19 | 23.87 | 89.06 | 101.88 | 137.75 | 47.7 | 29.825 | 2.221 |
| 20 | 23.87 | 89.06 | 98.38 | 132.00 | 46.8 | 30.75 | 2.23662 |
| 21 | 23.87 | 89.05 | 98.75 | 130.38 | 46.55 | 30.905 | 2.30141 |
| 22 | 23.86 | 89.05 | 90.63 | 121.25 | 43.95 | 31.515 | 2.36534 |
| 23 | 23.86 | 89.05 | 100.25 | 137.00 | 43.95 | 31.575 | 2.6504 |
| 24 | 23.86 | 89.05 | 98.63 | 135.38 | 45.6 | 30.325 | 2.69318 |
| 25 | 23.86 | 89.05 | 77.13 | 102.63 | 46.5 | 30.24 | 2.49691 |
| 26 | 23.86 | 89.05 | 110.63 | 149.50 | 46.1 | 30.565 | 2.45326 |
| 27 | 23.86 | 89.04 | 84.00 | 117.38 | 45.65 | 30.725 | 2.49773 |
| 28 | 23.86 | 89.04 | 87.00 | 120.00 | 47.75 | 29.385 | 2.46731 |
| 29 | 23.86 | 89.04 | 74.00 | 98.63 | 47.45 | 29.68 | 2.42191 |
| 30 | 23.86 | 89.04 | 93.88 | 128.88 | 46.25 | 29.75 | 2.43296 |
| 31 | 23.86 | 89.04 | 139.38 | 194.50 | 45.6 | 31.53 | 2.5824 |
| 32 | 23.86 | 89.03 | 71.63 | 110.50 | 48.35 | 29.695 | 2.54143 |
| 33 | 23.86 | 89.03 | 98.63 | 135.38 | 48.9 | 28.985 | 2.27062 |
| 34 | 23.86 | 89.03 | 91.50 | 135.25 | 50.4 | 29.13 | 2.27246 |

**Table S2(b):** Average values of measured Particulate Matter Concentrations in the residential area

| **FID** | **Latitude** | **Longitude** | **PM _2.5_ (µg/m^3^)** | **PM_10_(µg/m^3^)** | **Relative Humidity (%)** | **Temperature (^0^C)** | **Wind Speed (m/s)** |
| --- | --- | --- | --- | --- | --- | --- | --- |
| 0 | 23.88 | 89.09 | 123.88 | 172.25 | 63.05 | 22.05 | 2.66447 |
| 1 | 23.88 | 89.09 | 111.88 | 146.63 | 62.3 | 23.25 | 2.59965 |
| 2 | 23.88 | 89.09 | 117.75 | 156.13 | 57.3 | 24.24 | 2.56973 |
| 3 | 23.88 | 89.08 | 117.13 | 145.50 | 55.55 | 25.03 | 2.44217 |
| 4 | 23.88 | 89.08 | 119.50 | 166.25 | 54.35 | 25.72 | 2.41741 |
| 5 | 23.88 | 89.08 | 128.25 | 172.75 | 54.8 | 25.45 | 2.55066 |
| 6 | 23.87 | 89.08 | 120.75 | 167.38 | 56.25 | 25.04 | 2.59325 |
| 7 | 23.87 | 89.08 | 128.88 | 171.50 | 54.05 | 25.56 | 2.69027 |
| 8 | 23.87 | 89.08 | 124.63 | 161.25 | 55.25 | 25.97 | 2.73734 |
| 9 | 23.87 | 89.07 | 120.88 | 157.88 | 51.85 | 25.89 | 2.58315 |
| 10 | 23.87 | 89.07 | 117.50 | 160.50 | 49.85 | 26.57 | 2.41647 |
| 11 | 23.87 | 89.07 | 116.75 | 162.88 | 49.65 | 27.37 | 2.2601 |
| 12 | 23.87 | 89.07 | 126.50 | 175.25 | 49.1 | 26.90 | 2.17797 |
| 13 | 23.87 | 89.07 | 117.50 | 161.63 | 45.75 | 29.05 | 2.3887 |
| 14 | 23.87 | 89.07 | 126.25 | 165.75 | 46.25 | 29.02 | 2.46195 |
| 15 | 23.87 | 89.06 | 113.00 | 145.88 | 49.15 | 28.12 | 2.62836 |
| 16 | 23.87 | 89.06 | 100.50 | 147.63 | 48.5 | 27.85 | 2.3011 |
| 17 | 23.87 | 89.06 | 121.13 | 158.75 | 46.35 | 29.47 | 2.27733 |
| 18 | 23.86 | 89.06 | 109.13 | 140.13 | 47.65 | 29.49 | 2.29825 |
| 19 | 23.87 | 89.06 | 110.38 | 152.00 | 47 | 28.39 | 2.57818 |
| 20 | 23.86 | 89.05 | 109.13 | 147.13 | 49.7 | 27.75 | 2.6449 |
| 21 | 23.86 | 89.05 | 110.75 | 149.00 | 46.8 | 29.14 | 2.52846 |
| 22 | 23.87 | 89.05 | 99.00 | 132.75 | 47.45 | 29.29 | 2.75719 |
| 23 | 23.86 | 89.05 | 104.75 | 142.38 | 46.15 | 28.37 | 2.88389 |
| 24 | 23.86 | 89.05 | 88.88 | 126.38 | 43.25 | 29.97 | 2.86496 |
| 25 | 23.86 | 89.05 | 140.13 | 177.63 | 43.75 | 30.80 | 2.84656 |
| 26 | 23.86 | 89.04 | 165.38 | 223.38 | 45.05 | 30.94 | 2.86094 |
| 27 | 23.86 | 89.04 | 97.25 | 137.50 | 45.8 | 28.94 | 2.81283 |
| 28 | 23.86 | 89.04 | 102.00 | 137.25 | 48.85 | 28.85 | 2.80867 |
| 29 | 23.86 | 89.04 | 91.88 | 125.75 | 46.2 | 29.36 | 2.80976 |
| 30 | 23.86 | 89.04 | 111.88 | 150.88 | 43.8 | 31.18 | 2.78821 |
| 31 | 23.86 | 89.03 | 99.50 | 140.75 | 47.5 | 30.14 | 2.5517 |
| 32 | 23.86 | 89.03 | 83.50 | 115.25 | 46.7 | 30.32 | 2.29842 |

**Table S3:** Descriptive Statistics of the measure of particulate matter concentration

| Country/ Places | Total Sample | Minimum (µg/m^3^) | | Maximum (µg/m^3^) | | Average ± SD (µg/m^3^) | | References |
| --- | --- | --- | --- | --- | --- | --- | --- | --- |
|  |  | PM _2.5_ | PM _10_ | PM _2.5_ | PM _10_ | PM _2.5_ | PM _10_ |  |
| Beijing, China | - |  |  |  |  | 47.6±42.0 | 75.0±56.7 | Hu et al., 2022 |
| Bengaluru, India | - | - | - | - | - | 32.13±18.87 | 95.61±42.55 | Sowmya et al.,2024 |
| Karachi, Pakistan | - | - | - | - | - | 55.99 | 105.00 | Malhi et al.,2023 |
| Brazil | - | - | - | - | - | 10.6 ± 7.2 | 25±11 | Cruz et al., 2024 |
| New York | - | - | - | - | - | 25.4 ± 47.1 | 25.4 ± 47.1 | Lu et al., 2024 |
| South Africa | - | - | - | - | - | 106.38±55.36 | 189.95±103.54 | Barhadje et al., 2024 |
| Dhaka | 89 | 5.27 | - | 105 |  | 32.7±20.3 |  | Rahman et al.,2021 |
| Dhaka | - | - | - | - | - | 44.1 | 91.7 | Hoque et al.,2020 |
| Gazipur | - | 80.2 | 82 | 231.5 | 358 | - | - | Hoque et al., 2015 |
|  | | | | | | | | |
| Industrial Area | 35 | 71.63 | 98.63 | 139.38 | 194.50 | 93.26±14.007 | 128.35±21.61 | * This Study |
| Residential Area | 33 | 83.50 | 115.25 | 165.38 | 223.38 | 114.43±15.56 | 154.36±19.72 | * This Study |
| **Standards Values of PM (24h)** | | | | | | | | |
|  | | | PM _2.5_ | | | PM _10_ | |  |
| BD | | | 65 | | | 150 | | Hossen and Hoque, 2016 |
| WHO | | | 25 | | | 50 | | Hossen and Hoque, 2016 |

*BD = Bangladesh; WHO = World Health Organization; SD= Standard deviation

| **Sample no.**  **Table (S4-a):** Potential Health Risk (HQ) Analysis using measured PM Concentrations in the industrial areas | **PM _2.5_ (µg/m^3^)** | **PM_10_(µg/m^3^)** | **ADD(PM_2.5_)** | **ADD(PM_10_)** | **RfD (PM_2.5_)** | **RfD(PM_10_)** | **HQ(PM_2.5_)** | **HQ (PM_10_)** |
| --- | --- | --- | --- | --- | --- | --- | --- | --- |
| 1 | 71.63 | 98.63 | 19.54479 | 26.91247 | 9.55068 | 40.931 | 2.04643 | 0.657508 |
| 2 | 74.00 | 102.63 | 20.19288 | 28.00397 | 9.55068 | 40.931 | 2.114287 | 0.684175 |
| 3 | 77.13 | 106.13 | 21.04562 | 28.95904 | 9.55068 | 40.931 | 2.203573 | 0.707509 |
| 4 | 80.50 | 108.63 | 21.96658 | 29.64123 | 9.55068 | 40.931 | 2.300001 | 0.724176 |
| 5 | 80.50 | 109.75 | 21.96658 | 29.94822 | 9.55068 | 40.931 | 2.300001 | 0.731676 |
| 6 | 80.88 | 110.50 | 22.0689 | 30.15288 | 9.55068 | 40.931 | 2.310715 | 0.736676 |
| 7 | 81.75 | 113.25 | 22.30767 | 30.90329 | 9.55068 | 40.931 | 2.335715 | 0.755009 |
| 8 | 84.00 | 113.63 | 22.92164 | 31.00562 | 9.55068 | 40.931 | 2.400001 | 0.757509 |
| 9 | 84.00 | 114.25 | 22.92164 | 31.17616 | 9.55068 | 40.931 | 2.400001 | 0.761676 |
| 10 | 84.75 | 116.00 | 23.1263 | 31.6537 | 9.55068 | 40.931 | 2.42143 | 0.773343 |
| 11 | 85.13 | 116.63 | 23.22863 | 31.82425 | 9.55068 | 40.931 | 2.432144 | 0.77751 |
| 12 | 85.63 | 117.13 | 23.36507 | 31.96068 | 9.55068 | 40.931 | 2.44643 | 0.780843 |
| 13 | 86.25 | 117.38 | 23.53562 | 32.0289 | 9.55068 | 40.931 | 2.464287 | 0.78251 |
| 14 | 87.00 | 118.50 | 23.74027 | 32.33589 | 9.55068 | 40.931 | 2.485716 | 0.79001 |
| 15 | 87.25 | 120.00 | 23.80849 | 32.74521 | 9.55068 | 40.931 | 2.492858 | 0.80001 |
| 16 | 88.00 | 121.13 | 24.01315 | 33.05219 | 9.55068 | 40.931 | 2.514287 | 0.80751 |
| 17 | 90.63 | 121.25 | 24.72945 | 33.0863 | 9.55068 | 40.931 | 2.589287 | 0.808343 |
| 18 | 91.50 | 122.88 | 24.96822 | 33.52973 | 9.55068 | 40.931 | 2.614287 | 0.819177 |
| 19 | 92.13 | 127.50 | 25.13877 | 34.79178 | 9.55068 | 40.931 | 2.632144 | 0.850011 |
| 20 | 93.88 | 128.88 | 25.6163 | 35.16699 | 9.55068 | 40.931 | 2.682144 | 0.859177 |
| 21 | 94.00 | 130.13 | 25.65041 | 35.50808 | 9.55068 | 40.931 | 2.685716 | 0.867511 |
| 22 | 94.25 | 130.38 | 25.71863 | 35.5763 | 9.55068 | 40.931 | 2.692859 | 0.869177 |
| 23 | 97.50 | 132.00 | 26.60548 | 36.01973 | 9.55068 | 40.931 | 2.785716 | 0.880011 |
| 24 | 98.38 | 132.13 | 26.84425 | 36.05384 | 9.55068 | 40.931 | 2.810716 | 0.880844 |
| 25 | 98.63 | 133.63 | 26.91247 | 36.46315 | 9.55068 | 40.931 | 2.817859 | 0.890844 |
| 26 | 98.63 | 135.25 | 26.91247 | 36.90658 | 9.55068 | 40.931 | 2.817859 | 0.901678 |
| 27 | 98.75 | 135.38 | 26.94658 | 36.94068 | 9.55068 | 40.931 | 2.82143 | 0.902511 |
| 28 | 100.25 | 135.38 | 27.35589 | 36.94068 | 9.55068 | 40.931 | 2.864287 | 0.902511 |
| 29 | 101.00 | 137.00 | 27.56055 | 37.38411 | 9.55068 | 40.931 | 2.885716 | 0.913345 |
| 30 | 101.63 | 137.75 | 27.7311 | 37.58877 | 9.55068 | 40.931 | 2.903573 | 0.918345 |
| 31 | 101.88 | 138.50 | 27.79932 | 37.79342 | 9.55068 | 40.931 | 2.910716 | 0.923345 |
| 32 | 110.63 | 149.50 | 30.18699 | 40.79507 | 9.55068 | 40.931 | 3.160716 | 0.996679 |
| 33 | 117.88 | 178.75 | 32.16534 | 48.77671 | 9.55068 | 40.931 | 3.367859 | 1.191681 |
| 34 | 125.00 | 187.25 | 34.10959 | 51.09616 | 9.55068 | 40.931 | 3.57143 | 1.248349 |
| 35 | 139.38 | 194.50 | 38.03219 | 53.07452 | 9.55068 | 40.931 | 3.982145 | 1.296683 |

**Table (S4-b):** Potential Health Risk (HQ) Analysis using measured PM Concentrations in the residential areas

| **Sample no.** | **PM _2.5_ (µg/m^3^)** | **PM_10_(µg/m^3^)** | **ADD(PM_2.5_)** | **ADD(PM_10_)** | **RfD (PM_2.5_)** | **RfD(PM_10_)** | **HQ(PM_2.5_)** | **HQ (PM_10_)** |
| --- | --- | --- | --- | --- | --- | --- | --- | --- |
| 1 | 83.50 | 115.25 | 22.78521 | 31.44904 | 9.55068 | 40.931 | 2.385716 | 0.768343 |
| 2 | 88.88 | 125.75 | 24.25192 | 34.31425 | 9.55068 | 40.931 | 2.539287 | 0.838344 |
| 3 | 91.88 | 126.38 | 25.07055 | 34.48479 | 9.55068 | 40.931 | 2.625001 | 0.84251 |
| 4 | 97.25 | 132.75 | 26.53726 | 36.22438 | 9.55068 | 40.931 | 2.778573 | 0.885011 |
| 5 | 99.00 | 137.25 | 27.01479 | 37.45233 | 9.55068 | 40.931 | 2.828573 | 0.915011 |
| 6 | 99.50 | 137.50 | 27.15123 | 37.52055 | 9.55068 | 40.931 | 2.842859 | 0.916678 |
| 7 | 100.50 | 140.13 | 27.42411 | 38.23685 | 9.55068 | 40.931 | 2.87143 | 0.934178 |
| 8 | 102.00 | 140.75 | 27.83342 | 38.4074 | 9.55068 | 40.931 | 2.914287 | 0.938345 |
| 9 | 104.75 | 142.38 | 28.58384 | 38.85082 | 9.55068 | 40.931 | 2.992859 | 0.949178 |
| 10 | 109.13 | 145.50 | 29.77767 | 39.70356 | 9.55068 | 40.931 | 3.117859 | 0.970012 |
| 11 | 109.13 | 145.88 | 29.77767 | 39.80589 | 9.55068 | 40.931 | 3.117859 | 0.972512 |
| 12 | 110.38 | 146.63 | 30.11877 | 40.01055 | 9.55068 | 40.931 | 3.153573 | 0.977512 |
| 13 | 110.75 | 147.13 | 30.2211 | 40.14699 | 9.55068 | 40.931 | 3.164287 | 0.980845 |
| 14 | 111.88 | 147.63 | 30.52808 | 40.28342 | 9.55068 | 40.931 | 3.19643 | 0.984179 |
| 15 | 111.88 | 149.00 | 30.52808 | 40.65863 | 9.55068 | 40.931 | 3.19643 | 0.993346 |
| 16 | 113.00 | 150.88 | 30.83507 | 41.17027 | 9.55068 | 40.931 | 3.228573 | 1.005846 |
| 17 | 116.75 | 152.00 | 31.85836 | 41.47726 | 9.55068 | 40.931 | 3.335716 | 1.013346 |
| 18 | 117.13 | 156.13 | 31.96068 | 42.60288 | 9.55068 | 40.931 | 3.34643 | 1.040846 |
| 19 | 117.50 | 157.88 | 32.06301 | 43.08041 | 9.55068 | 40.931 | 3.357145 | 1.052513 |
| 20 | 117.50 | 158.75 | 32.06301 | 43.31918 | 9.55068 | 40.931 | 3.357145 | 1.058346 |
| 21 | 117.75 | 160.50 | 32.13123 | 43.79671 | 9.55068 | 40.931 | 3.364287 | 1.070013 |
| 22 | 119.50 | 161.25 | 32.60877 | 44.00137 | 9.55068 | 40.931 | 3.414287 | 1.075013 |
| 23 | 120.75 | 161.63 | 32.94986 | 44.1037 | 9.55068 | 40.931 | 3.450002 | 1.077513 |
| 24 | 120.88 | 162.88 | 32.98397 | 44.44479 | 9.55068 | 40.931 | 3.453573 | 1.085847 |
| 25 | 121.13 | 165.75 | 33.05219 | 45.22932 | 9.55068 | 40.931 | 3.460716 | 1.105014 |
| 26 | 123.88 | 166.25 | 33.8026 | 45.36575 | 9.55068 | 40.931 | 3.539288 | 1.108347 |
| 27 | 124.63 | 167.38 | 34.00726 | 45.67274 | 9.55068 | 40.931 | 3.560716 | 1.115847 |
| 28 | 126.25 | 171.50 | 34.45068 | 46.79836 | 9.55068 | 40.931 | 3.607145 | 1.143347 |
| 29 | 126.50 | 172.25 | 34.5189 | 47.00301 | 9.55068 | 40.931 | 3.614288 | 1.148348 |
| 30 | 128.25 | 172.75 | 34.99644 | 47.13945 | 9.55068 | 40.931 | 3.664288 | 1.151681 |
| 31 | 128.88 | 175.25 | 35.16699 | 47.82164 | 9.55068 | 40.931 | 3.682145 | 1.168348 |
| 32 | 140.13 | 177.63 | 38.23685 | 48.46973 | 9.55068 | 40.931 | 4.003573 | 1.184181 |
| 33 | 165.38 | 223.38 | 45.12699 | 60.95384 | 9.55068 | 40.931 | 4.725002 | 1.489185 |

**Table S5.** Classification of atmospheric stability (A-F) with corresponding descriptions, atmospheric conditions, and their effects on pollutant dispersion (adapted from Mitchell Jr. & Timbre, 1979; U.S. Environmental Protection Agency, 2000).

| **Class** | **Description** | **Atmospheric Condition** | **Effect on Dispersion** |
| --- | --- | --- | --- |
| A | Very Unstable | Strong solar radiation (sunny, hot day with light winds) | Very strong vertical and horizontal mixing - pollutants spread quickly and widely. |
| B | Unstable | Sunny day with moderate winds | Strong mixing, good dispersion, pollutants dilute faster. |
| C | Slightly Unstable | Partly cloudy day or weak solar radiation | Moderate turbulence, moderate dispersion. |
| D | Neutral | Overcast day or night with moderate/strong winds | Mechanical mixing dominates - typical urban conditions; steady dispersion. |
| E | Slightly Stable | Clear night with light winds | Limited turbulence - pollutants spread slowly, concentration remains higher near the source. |
| F | Very Stable | Clear, calm night (temperature inversion) | Very poor mixing - pollutants stay concentrated near the ground, higher risk of dangerous concentrations. |

**Table S6.** Gaussian Dispersion Model based evaluation of Travelling Distances of the Particulate Matter from the Source Points

| **Source Points** | **Dispersion of PM 2.5 (m)** |  | **Dispersion of PM 10 (m)** |
| --- | --- | --- | --- |
| 1 | 1257.7 |  | 1860.2 |
| 2 | 1259.4 |  | 1606.9 |
| 3 | 1273.0 |  | 1501.0 |
| 4 | 1302.0 |  | 1890.3 |
| 5 | 1306.6 |  | 1506.2 |
| 6 | 1313.3 |  | 1580.7 |
| 7 | 1317.4 |  | 1434.5 |
| 8 | 1340.7 |  | 1482.4 |
| 9 | 1356.7 |  | 1634.5 |
| 10 | 1367.0 |  | 1465.9 |
| 11 | 1374.2 |  | 1610.9 |
| 12 | 1380.4 |  | 1603.9 |
| 13 | 1380.8 |  | 1614.5 |
| 14 | 1386.0 |  | 1586.1 |
| 15 | 1397.8 |  | 1657.9 |
| 16 | 1397.8 |  | 1640.5 |
| 17 | 1409.3 |  | 1717.4 |
| 18 | 1413.8 |  | 1626.6 |
| 19 | 1415.8 |  | 1649.9 |
| 20 | 1420.6 |  | 1821.7 |
| 21 | 1421.3 |  | 1779.8 |
| 22 | 1422.9 |  | 1746.0 |
| 23 | 1444.7 |  | 1666.2 |
| 24 | 1453.5 |  | 1672.7 |
| 25 | 1458.3 |  | 1651.0 |
| 26 | 1481.4 |  | 1502.2 |
| 27 | 1487.8 |  | 1806.7 |
| 28 | 1521.9 |  | 1599.7 |
| 29 | 1532.9 |  | 1625.6 |
| 30 | 1541.7 |  | 1495.7 |
| 31 | 1550.8 |  | 1692.0 |
| 32 | 1569.0 |  | 1994.8 |
| 33 | 1573.3 |  | 1542.4 |
| 34 | 1581.6 |  | 1788.3 |
| 35 | 1706.9 |  | 1786.9 |

**Table S7.** Country Life-Table Dataset for AirQ+ Model: Population Exposure and Baseline Incidence Rates of Health Outcomes Associated with PM₂.₅ and PM₁₀ in Industrial and Residential Areas

| **Location** | **Total Population** | **Health Outcomes** | **Exposed Population for PM_2.5_** | | **Exposed Population for PM_10_** | |
| --- | --- | --- | --- | --- | --- | --- |
|  |  |  | **At risk population**  **(Age Group)** | **Baseline incidence (per 1000,000 population)** | **At risk population**  **(Age Group)** | **Baseline incidence (per 1000,000 population)** |
| **Industrial**  **Area** | 20000 | ALRI | -- | 1.65 | - | - |
|  |  | COPD | 20000 (30+) | 6.29 | - | - |
|  |  | IHD | 20000 (30+) | 17.46 | 20000 (30+) | 17.46 |
|  |  | LC | 20000 (30+) | 1.30 | 20000 (30+) | 1.30 |
|  |  | Stroke | 20000 (30+) | 15.73 | - | - |
| **Residential**  **Area** | **104833** | ALRI | 8387 (<5 years) | 1.65 | - | - |
|  |  | COPD | 57658 (30+) | 6.29 | - | - |
|  |  | IHD | 57658 (30+) | 17.46 | 57658 (30+) | 17.46 |
|  |  | LC | 57658 (30+) | 1.31 | 57658 (30+) | 1.30 |
|  |  | Stroke | 57658 (30+) | 15.73 | - | - |
| ALRI: Acute lower respiratory infections; COPD: Chronic obstructive pulmonary disease; IHD: Ischemic heart disease; LC: Lung cancer | | | | | | |

**References:**

Hossen, M.A. and Hoque, A. (2016). Variation of ambient air quality scenario in Chittagong City: A case study of air pollution.

Mitchell Jr, A. E., & Timbre, K. O. (1979, June). Atmospheric stability class from

horizontal wind fluctuation. In 72nd Annual Meeting of Air Pollution Control Association, Cincinnati, OH.

U.S. Environmental Protection Agency. (2000). Meteorological monitoring guidance for

regulatory modeling applications (EPA-454/R-99-005). U.S. Environmental Protection Agency.
